# Fluorescence Discrimination of Cancer from Inflammation by Selective Targeting Folate Receptor α

**DOI:** 10.1021/cbmi.5c00087

**Published:** 2025-10-02

**Authors:** Yunlong Li, Nida El Islem Guissi, Junming Dong, Sunil Singhal, Bo Dai, Christopher Butch, Huiming Cai, Yiqing Wang

**Affiliations:** † Department of Biomedical Engineering, College of Engineering and Applied Sciences, State Key Laboratory of Analytical Chemistry for Life Science, 12581 Nanjing University, Nanjing 210023, China; ‡ Departments of Radiation Oncology, Medicine and Surgery, 6569 University of Pennsylvania, Philadelphia, Pennsylvania 19104, United States; § Department of Thoracic Surgery, Nanjing Drum Tower Hospital, The Affiliated Hospital of Nanjing University Medical School, Nanjing 210008, China; ∥ Nanjing Nuoyuan Medical Devices Co. Ltd, Nanjing 211500, China

**Keywords:** fluorescent probe, image guided surgery, folate receptor α&β, tumor-targeting, inflammation

## Abstract

We present NY-07, an antifolate dye conjugate capable of intrasurgical discrimination between inflammatory and cancerous tissues. This capability addresses a longstanding problem in fluorescence-guided surgery, where the current FDA-approved probes exhibit false-positive rates of 25–68% due to nonspecific accumulation in inflammatory tissue, leading to unnecessary tissue resection and increased surgical morbidity. NY-07 selectively targets folate receptor α (FRα) overexpressed in tumors while exhibiting 8-fold reduced affinity for folate receptor β (FRβ) predominantly found on inflammatory macrophages (*K*_d_ = 61.67 nM vs 486.9 nM). In mouse models, NY-07 achieved a tumor-to-background ratio of 3.23 ± 0.28 with fluorescence signals in tumor tissue significantly exceeding inflammatory areas (*p* < 0.01). The probe detected submillimeter cancer lesions while avoiding false signals in pneumonia and arthritis models. Co-localization studies using immunohistochemical staining and fluorescence microscopy confirmed that FRα-positive areas in tumor tissue exhibited strong fluorescence intensity, while FRβ-positive areas showed a minimal signal. NY-07 maintained diagnostic imaging windows exceeding 12 days and demonstrated acceptable safety profiles in Phase I clinical trials (CTA: CXHL2401187), having received IND approval from both FDA and NMPA. These results position NY-07 as a strong clinical candidate with the potential to improve surgical precision and reduce false-positive resections.

## Graphical abstract

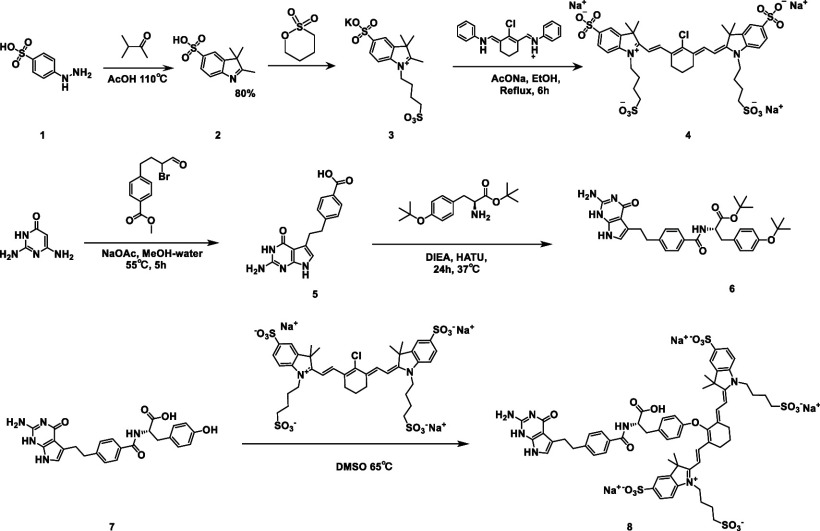

## Introduction

Fluorescence-guided surgery (FGS) has emerged as a transformative tool for real-time intraoperative diagnosis, with advances in molecular imaging enabling precise visualization of tumor margins, submillimeter lesions, and lymph node metastases.^1–6^ Despite these advances, a critical challenge persists, reliably distinguishing malignant tissue from inflammatory foci during surgery.^7^ This difficulty is heightened by the close association between cancer and inflammation, with inflammation frequently colocalizing with tumors and contributing to tumorigenesis.^8^ Conventional probes accumulate in inflammatory sites, generating nonspecific fluorescence that limits their diagnostic value. In these situations, surgeons fall back on visual inspection and palpation and often adopt a conservative strategy, treating suspicious inflammatory lesions as malignant to avoid residual tumor tissues.^9–11^ While this approach safeguards against under-resection, it also drives false positives and unnecessary tissue removal, increasing the risk of postoperative complications such as pneumothorax, hemorrhage, and pyothorax^12,13^ (Scheme 1).

**Scheme 1:**
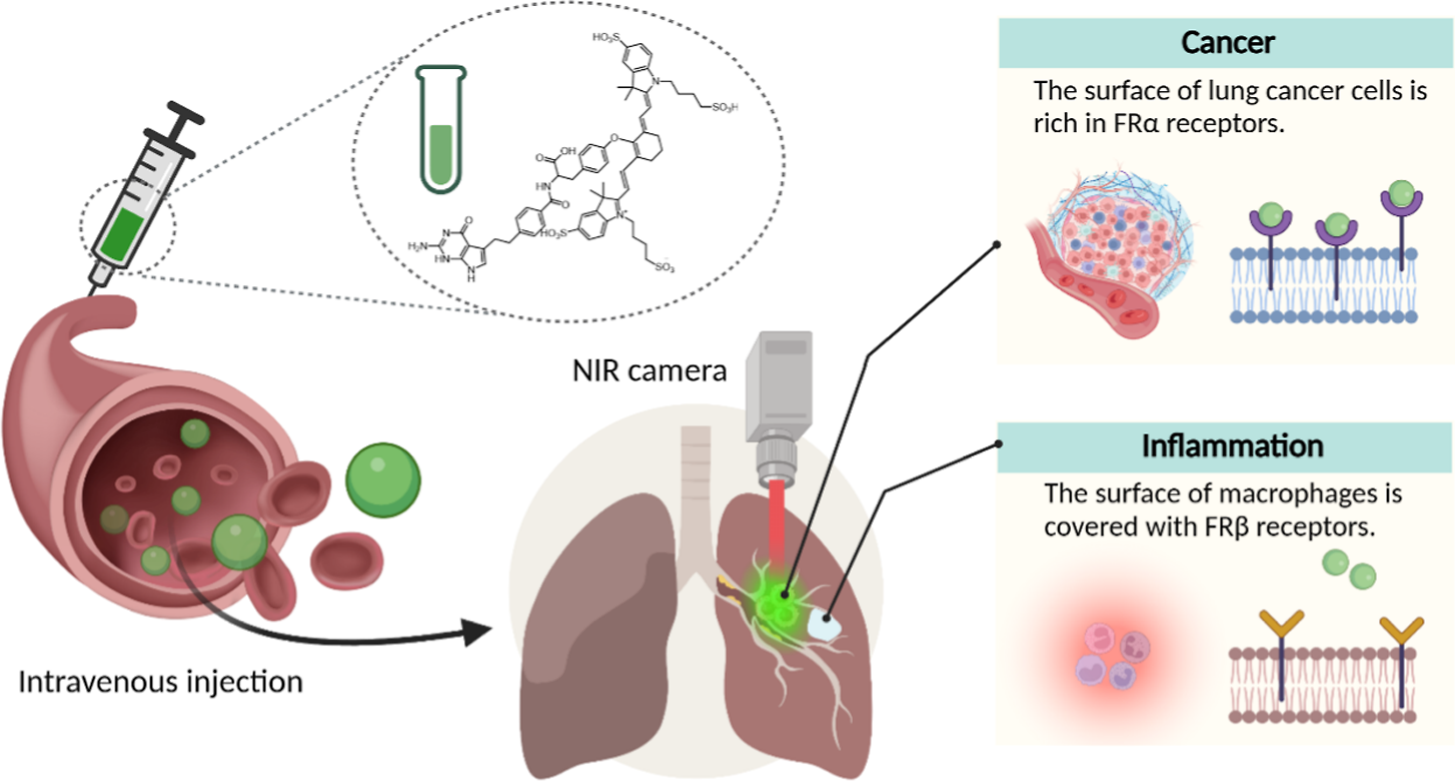
NY-07 is Circulated Throughout the Body by Intravenous Injection. Tumor Cells Overexpress Folate Receptor α on the Surface and Specifically Recognize NY-07 and Endocytose It after Binding, While Macrophages in Inflammatory Tissues Overexpress Folate Receptor β and Do Not Specifically Recognize NY-07, and Tumor Sites Show High Fluorescence Under NIR Camera Irradiation to Distinguish Tumor From Inflammatory Tissues

The nonspecificity of the current probes arises from their mechanisms of accumulation at tumor sites. For example, indocyanine green (ICG) localizes to tumors via the enhanced permeability and retention (EPR) effect, with similar accumulation in tumor and inflamed tissues due to shared vascular alterations in the diseased microenvironment.^14,15^ Similarly, OTL-38, an FDA-approved folate receptor (FR)–targeted near-infrared fluorescent probe, has demonstrated promising clinical efficacy but still produces a 32.7% false-positive rate in ovarian and lung cancer surgeries, primarily because it cannot distinguish tumors from inflammation.^16,17^ Other tumor-associated markers such as CXCR4,^18^ αvβ3,^19^ and COX-2 ^20^ face the same limitation, as these receptors are also expressed in both tumor and inflammatory tissues. Identifying a receptor that is differentially expressed between tumors and inflammation therefore remains an urgent priority for improving probe selectivity.

While folate receptors have long been explored for cancer imaging, as exemplified by OTL-38, improved tumor-inflammation discrimination may be achieved by exploiting the distinct biology of the α and β subtypes. FRα is upregulated in ∼40% of cancers,^21,22^ whereas FRβ is primarily expressed on activated inflammatory macrophages.^23^ However, previously reported folate-conjugated probes have shown comparable affinity for both subtypes, resulting in elevated false-positive signals.^24,25^ We therefore hypothesized that re-engineering folate-based probe architecture could enable selective FRα targeting to distinguish tumors from inflammation. Preclinical studies support this approach, showing that specific folate structures can preferentially bind FRα over FRβ.^26,27^ Guided by these findings, we designed NY-07, a pemetrexed-derived probe optimized for FRα selectivity.

Consistent with the pemetrexed parent compound, NY-07 demonstrated high binding selectivity for FRα, with a dissociation constant (*K*_d_) of 61.67 nM compared to 486.9 nM for FRβ. In vivo imaging showed a tumor-to-background ratio (TBR) of 3.23 ± 0.28 at 1 h post administration, meeting clinical imaging thresholds and maintaining an extended imaging window (TBR >1.5) for up to 12 days. The probe enabled specific targeting across multiple tumor types and detected lesions smaller than 1 mm, supporting intraoperative surgical decision-making. In inflammation models (peritonitis and pneumonia), tumor fluorescence intensity (3.57 ± 0.76 photons/sec/cm^2^/sr × 10^8^) was significantly higher than in inflamed tissues (1.35 ± 0.16; *P* < 0.01, Student’s *t*-test). Together, these findings confirm NY-07’s high FRα specificity is maintained in vivo and highlight its translational potential for reducing false-positive rates and enabling precise tumor-inflammation discrimination in FGS.

## Experimental Section

### Synthesis of NY-07 and Structural Characterization and Performance Testing

Synthetic methods and ^1^HNMR and MS characterizations are described in the Supporting Information. Absorption spectra and emission spectra of NY-07 were tested using in phosphate buffer solution (PBS) (pH = 7.4). A fluorescence intensity of 1 nM NY-07, S0456, ICG, and OTL-38 was tested in PBS. Stability of NY-07 and OTL-38 experiments was performed in PBS solution at a concentration of 1 nM.

### Computational Studies of NY-07, OTL-38, Folic acid, and Pemetrexed

NY07 was identified through a computational screening approach targeting selective folate receptor inhibition. Regression models were trained on data sets of known inhibitors for folate receptor alpha (FRα, CHEMBL2121) and folate receptor beta (FRβ, CHEMBL5064) retrieved from the ChEMBL database.

The trained models were subsequently employed to predict the inhibitory activity of a library of modified approved antifolates against both receptor subtypes. Structural modifications included systematic amino acid substitutions and the incorporation of truncated linker moieties into existing antifolate scaffolds. For each compound, the predicted IC_50_ values for FRα and FRβ were used to calculate the selectivity ratio (FRβ IC_50_/FRα IC_50_), with higher ratios indicating preferential inhibition of FRα over FRβ.

The favorable predicted selectivity profile for NY-07 was subsequently confirmed through molecular docking studies. Based on the crystal structure of folate receptor alpha bound with folic acid (PDB ID: 4LRH), we performed molecular docking to evaluate NY07 alongside control compounds (OTL-38, folic acid, and pemetrexed). The receptor was prepared by removing the original ligand and minimizing the structure using Chimera’s Dock Prep. Ligands were generated from SMILES using RDKit, with 2D to 3D conversion (embedding: maxAttempts = 100, randomSeed = 0xf00d) and UFF optimization (maxIters = 1000). Open Babel was used to convert file formats and assign the MMFF94 charges. Docking was conducted using SMINA (seed = 0, autobox based on the original ligand, exhaustiveness = 24).

### Cell Culture

NCI-H1299, A549, RAW 264.7, SKOV-3, MDA-MB-231, and 293T cell lines were obtained from Procell (Wuhan, China) and grown using IMDM (KeyGEN BioTECH, Nanjing) containing 10% dialyzed fetal bovine serum (KeyGEN BioTECH, Nanjing) and 1% penicillin–streptomycin (KeyGEN BioTECH, Nanjing) in 5% CO_2_ atmosphere at 37 °C.

### FRα FRβ Affinity Assay

Seed 10^6^ 293T cells (within 10 passages) into each well of a six-well plate, add 2 mL of complete medium per well, and incubate for 24 h. Subsequently, aspirate the complete medium and replace it with 900 μL prewarmed Opiti-MEM per well. Prepare the transfection mixture by mixing 4.5 μL Lipofectamine 2000 with 45.5 μL Opti-MEM, incubating at room temperature for 5 min, combining it with a solution containing 3 μL (1 μg/mL) of FRα/FRβ target plasmid DNA and 47 μL Opti-MEM, and then mixing well and incubating for 15–18 min. Add the plasmid mixture to the cells in the 6-well plate, incubate at 37 °C for 5–6 h, and then replace the medium with 2 mL of prewarmed complete culture medium per well. 48 h post-transfection, collect the cells and reseed them into a 96-well plate at a density of 1 × 10^5^ cells per well (90 μL serum-free medium per well). Dilute the test compound with PBS to the desired concentration and add 5 μL per well, dilute ^3^H-folic acid with PBS, and add 5 μL per well to the wells with the compound, while adding 5 μL of PBS to blank wells. After incubating at 37 °C for 2 h, transfer the cells to Filtermat A using a cell harvester, wash them 3 times with precooled PBS and dry, transfer Filtermat A to a sample bag, add scintillation fluid, seal the bag, and finally measure the samples with a liquid scintillation counter.

### Western Blot

The protein expression levels of FRα and FRβ in NCI-H1299, A549, and RAW264.7 cell lines were determined by Western blot; after protein extraction, electrophoresis, and membrane transfer, the corresponding FRα FRβ mAb and secondary antibody were incubated, enhanced chemiluminescence (ECL) was used for color development, G:BOX chemiXR5 imaging was used for detection, and grayscale was analyzed using ImageJ software. In this assay, GAPDH was used as an internal reference.

### Cellular Uptake Assay

The cell imaging experiments were performed using a 20 mm confocal dish with NCI-H1299, A549, and RAW264.7 cells of 1.5 × 10^4^ cells/well. For cell imaging, NCI-H1299, A549, and RAW264.7 cells were first cultured for 24 h. And then, 100 nM NY-07 in DMEM was used to culture the cells for 2 h. After rinsing with PBS, 4% paraformaldehyde was added to confocal dish to fix cell. Cellular uptake was imaged using a laboratory-made near-infrared fluorescence microscopy.

### Competitive Cellular Uptake in the Presence of Inhibitors

For competitive inhibition experiments, the NCI-H1299 cell line was incubated with 100-fold folic acid and inhibitors (deoxyglucose + NaN_3_) for half an hour. Additionally, NaN_3_ combined with Deoxyglucose was used as an ATP consumption inhibitor to inhibit metabolic activities requiring ATP, confirming that NCI-H1299 enters cells through an active, energy-dependent uptake process. 100 nM NY-07 in DMEM without FBS were used to culture the cells for 2 h. After rinsing with PBS, 4% paraformaldehyde was added to the confocal dish to fix the cells. Fluorescence images were acquired using a laboratory-made near-infrared fluorescence microscopy.

### Specificity Test of NY-07 and OTL-38 on FRβ Overexpression RAW264.7 Cell Lines

For cell imaging, RAW264.7 cells were first cultured for 24 h. And then, 100 nM NY-07 and OTL-38 in DMEM were used to culture the cells for 2 h, respectively. After rinsing with PBS, 4% paraformaldehyde was added to the confocal dish to fix the cells. Cellular uptake was imaged using a laboratory-made near-infrared fluorescence microscopy.

### Cellular Toxicity Assay

The cytotoxicity of NY-07 to L02 cells was evaluated by using the MTT assay. A total of 10,000 cells were added to the complete medium in a 96-well plate, adjusting the total volume to 100 μL per well. Serial 10-fold dilutions of the drug, ranging from 1 mM to 1 nM, were prepared. The plates are incubated for 24, 48, and 72 h, respectively, at 37 °C in an atmosphere of 5% CO2. At the end of the incubation, the cell viability was determined using the MTT assay.

### Animal Model Establishment and Imaging

#### Animals

Animal experiments were conducted using 5 week-old BALB/c nude (*n* = 51) and BALB/c mice (*n* = 3) (18–20 g) purchased from Nanjing GemPharmatech. The animals were housed in a Specific Pathogen Free environment. The animal experimental protocol was reviewed and approved by the Animal Ethical and Welfare Committee (AEWC) and the Institutional Animal Care and Use Committee (IACUC) of Nanjing University (approval no. IACUC-2403007).

### Subcutaneous Tumor Xenograft Model Establishment and Imaging

Animals were laid flat on the experiment bench after they were anesthetized. 100 μL of PBS containing 10^6^ NCI-H1299 or A549 or SKOV-3 or MDA-MB-231 cells was subcutaneously injected into the armpit, and tumor growth was observed every 2 days. NCI-H1299 (*n* = 3) and A549 (*n* = 3) groups, and SKOV-3 and MDA-MB-231 groups, were injected with 10 nmol NY-07 via the tail vein and imaged using the IVIS imaging system 24 h later. When the tumor volume reached 200 mm^3^, BALB/c nude was injected with 10 nmol of NY-07 and anesthetized with isoflurane for imaging using IVIS Imaging System (PerkinElmer Inc., MA).

### Orthotopic Colorectal Mice Model Establishment and Imaging

A single intraperitoneal injection of 10% AOM, with an injection dose of 10 mg/kg, and 2.5% DSS drinking water were used for seven consecutive days; then, sterile distilled water was used for 7 days, and this cycle was repeated three times. Two mice were randomly killed to observe the pathological changes of colon tissue to confirm whether the model was successful. Each mouse was injected with 10 nmol of NY-07 through the tail vein (*n* = 3). BALB/c mice were euthanized 12 h later, and the colorectal tissue was removed and placed on a black board. FLI-10B (Nuoyuan Medical, Nanjing) was used to image the BALB/c colorectum. ImageJ and origin were used to quantitatively analyze the fluorescence intensity of the image.

### Orthotopic Liver Mice Model Establishment and Imaging

The density of MDA-MB-231 cells was adjusted to a 10^7^/mL cell suspension. After the BALB/c nude mice were anesthetized with isoflurane, the abdomen was disinfected, the median muscle layer was cut open, and the liver was exposed. 100 μL of MDA-MB-231 cell suspension was inoculated into the left lobe of the liver. The needle was kept in place for about 5 s and slowly withdrawn. The liver was wiped with an alcohol cotton swab, and the mouse was sutured. After being observed until it recovered normal breathing, it was taken to the mouse cage. Each mouse was injected with 10 nmol NY-07 via the tail vein. BALB/c nude mice were euthanized 24 h later, and the abdomen was opened to expose the liver tissue. All organs were imaged using FLI-10B (Nuoyuan Medical, Nanjing), and the fluorescence intensity was quantitatively analyzed using ImageJ and GraphPad Prism 9.0 software.

### Subcutaneous Chronic Inflammation Model Establishment and Imaging

NCI-H1299 tumor-bearing BALB/c nude mice were selected. After anesthesia, lipopolysaccharide (LPS) (3 μg/g body weight in PBS) was injected subcutaneously around the tumor every 2 days for a total of three injections. On the seventh day, the absence of obvious inflammatory reactions such as redness, swelling, pus, or exudate at the injection site indicated successful establishment of chronic inflammation. The model was divided into two groups: NY-07 (*n* = 3) group and the OTL-38 group (*n* = 3). Each BALB/c nude was injected with 10 nmol of NY-07 or OTL-38 through the tail vein, and images were taken 96 h later using an IVIS Imaging System (PerkinElmer Inc., MA). The fluorescence intensity of tumor and inflammatory areas was quantitatively analyzed using GraphPad Prism 9.0 software.

### Pneumonia Model Establishment and Imaging

After anesthesia, the mouse was placed in a supine position and secured to an operating board. The trachea was intubated with an intravenous needle, and 100 μL of 1.5 mg/mL LPS solution was instilled into the trachea. After instillation, the upright operating board was immediately rotated left and right to promote the distribution of LPS into the left and right lung tissues. The pneumonia model was later confirmed by IHC sectioning. Pneumonia models (*n* = 3) were intravenously administered NY-07 (10 nmol per) (*n* = 3), OTL38 (10 nmol per) (*n* = 3) and ICG (4 mg/kg) (*n* = 3) via the tail vein. 12 h later, the mice were euthanized, the lungs were removed, and the expression levels of inflammatory FRβ in the lungs were analyzed by IHC. The uptake of each probe was detected using near-infrared fluorescence microscopy.

### Determination of the NY-07 Tumor-to-Background Ratio

The imaging analysis method was consistent across all experiments, with tumor-to-background ratio (TBR) values calculated by comparing the fluorescence of the tumor region of interest (ROI) against the average fluorescence from three background ROI regions of equivalent size—two near to and one distant from the tumor. The mean TBR of the NY-07 group was expressed as the form of mean ± SD (*n* = 3) from independent experiments.

### Mouse Model of Carrageenan Induce Arthritis

30 μL of carrageenan mixture (1%, m/v) was injected into the distal end of the toe. 1 day later, the mixture of the same concentration and volume was injected again to construct a toe inflammation model. 10 nmol/per NY-07 (*n* = 3) and OTL-38 (*n* = 3) were injected through the tail vein, and real-time in vivo detection was performed using the IVIS imaging system on the second day.

### Persistence of NY-O7, OTL-38, and ICG Tumor Imaging

NCI-H1299 tumor-bearing mice (*n* = 1) were selected and injected with 10 nmol/mouse NY-07, OTL-38, and ICG probes, respectively. FLI-10B imaging was used on day 6 for the ICG group and on day 12 for NY-07, the OTL-38 group.

### Statistical Analysis

Living Image 4.0 software and ImageJ were used to quantify fluorescence intensity. For groups with *n* = 3, mean ± standard deviation (SD) was used to represent the values. Student’s *t*-test was used to compare whether the difference between the two means was significant or not. Origin 2023b or GraphPad Prism 9.0 software were used to analyze the data. *represents *p* < 0.05, **represents *p* < 0.01, ***represents *p* < 0.001.

## Results and Discussion

### Design Concept

The synthesis route of probe NY-07 is shown in Figure 1. The structures of compounds 2, 3, 4, 7, and 8 were characterized by ^1^H NMR (Figures S1–S5). The results of HPLC showed that the purity of the NY-07 compound was above 97.7% (Figure S6). The MS spectrum of NY07 is shown in Figure S7. The maximum absorption wavelength of NY-07 was 774 nm, and the maximum emission wavelength was 793 nm (Figure S8A). The fluorescence intensity of NY-07 was higher than those of S0456 and ICG at the same concentration (Figure S8B). This result not only confirmed that the fluorescence performance of NY-07 was superior to that of ICG but also demonstrated that replacing the glutamic acid moiety of pemetrexed with the tyrosine moiety not only formed part of the FRα binding ligand but also contributed to the delocalized electron system of the NIR dye, thereby increasing its fluorescence. The bright fluorescence of phenoxyvinyl ether-bridged cyanine dyes avoids the formation of unwanted byproducts and thus improves the stability (Figure S9). A computational docking study of OTL-38, NY-07, folic acid, and pemetrexed using the crystal structure of FRα (PDB code: 4LRH) demonstrated that these four compounds share the same binding mode within the FRα binding pocket. Specifically, the pteroyl group is partially buried within the binding cleft, forming strong hydrogen bonds with Asp81, Ser174, Arg106, Arg103, and His135. Additionally, the α-carboxylate of tyrosine interacts with Trp138, Trp140, and Gly137 of FRα, while the phenol of tyrosine is prominent, allowing room for the attachment of the NIR dye (Figure S10). Since the NIR dye is partially exposed to the solvent, its attachment is not expected to affect the binding affinity of for FRα.

**Figure 1:**
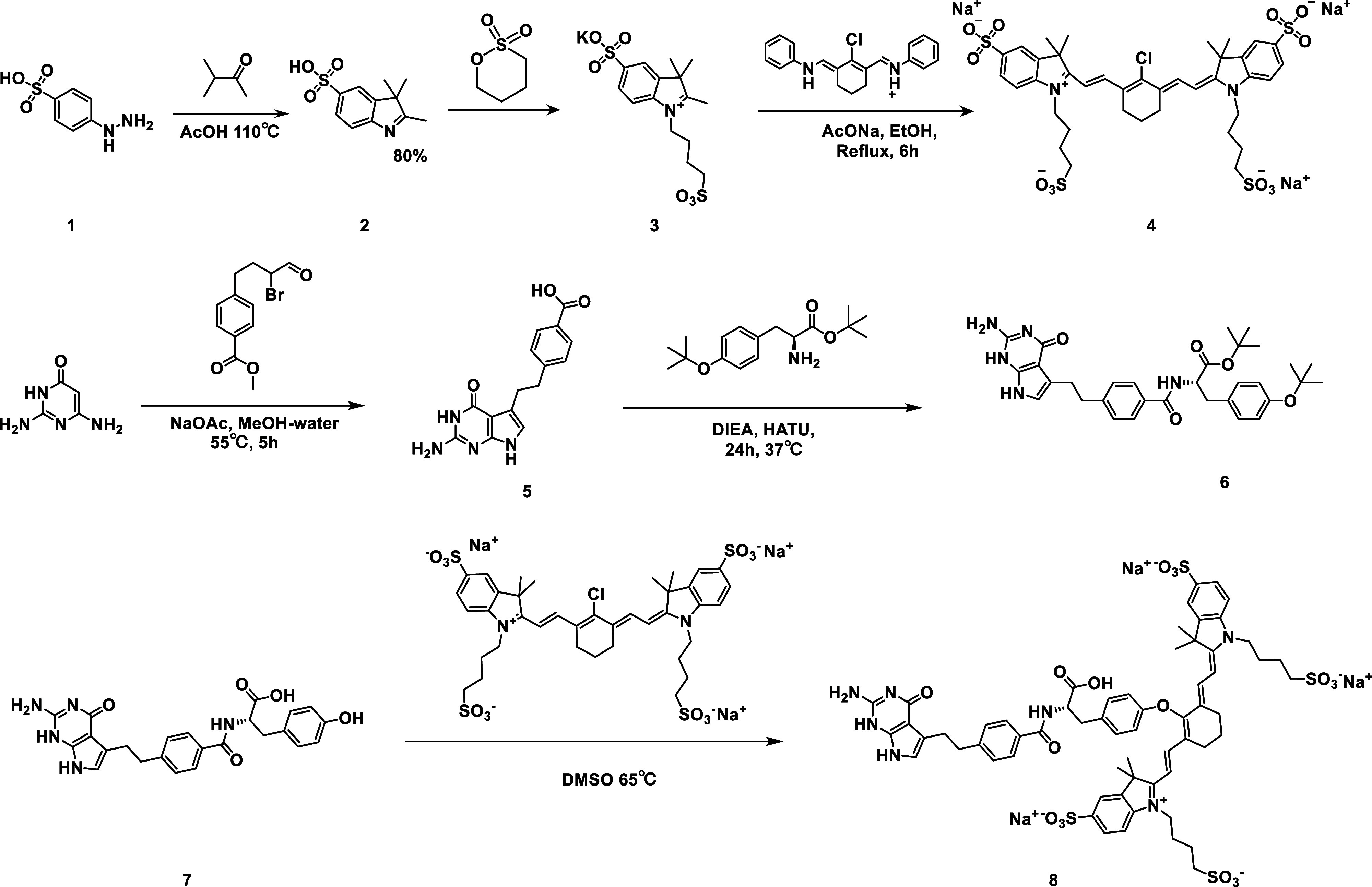
Synthesis route for NY-07.

### Evaluation of In Vitro Efficacy and Specificity of NY-07

To assess the in vitro FRα-specific targeting ability of the probe, three different cell lines were selected based on their intrinsic FRα FRβ expression levels. Western blot analysis revealed that FRα expression was the highest in NCI-H1299 cells compared with A549 and RAW264.7 cells. In contrast, FRβ expression was highest in RAW264.7 cells (Figure 2A,B). Fluorescence was observed throughout the cytoplasm of FRα positive (FRα^+^) NCI-H1299 cells, but it was absent in FRα negative (FRα^–^) A549 cells. Furthermore, uptake was competitively inhibited by excess folate in NCI-H1299 cells, indicating that the uptake by NCI-H1299 cells is FRα mediated. We also examined whether NY-07 could be endocytosed by FRβ expressing macrophages (Figure 2C). OTL-38 for FRβ was detected in the RAW264.7 cytoplasm its affinity for FRβ, whereas no fluorescence was observed for the NY-07 group (Figure 2D). This result suggested that due to the specificity of NY-07 for FRα, FRβ in inflammatory areas would not take up NY-07 and generate a false-positive signal. To further quantify their affinity for FRα and FRβ, three molecules were selected for competitive isotope assays. The experimental results showed that the affinity of NY-07 for FRα is 61.67 nM, while its affinity for FRβ is 486.9 nM, indicating that NY-07 exhibits significant selectivity for FRα over FRβ (Figure 2E,F).

**Figure 2:**
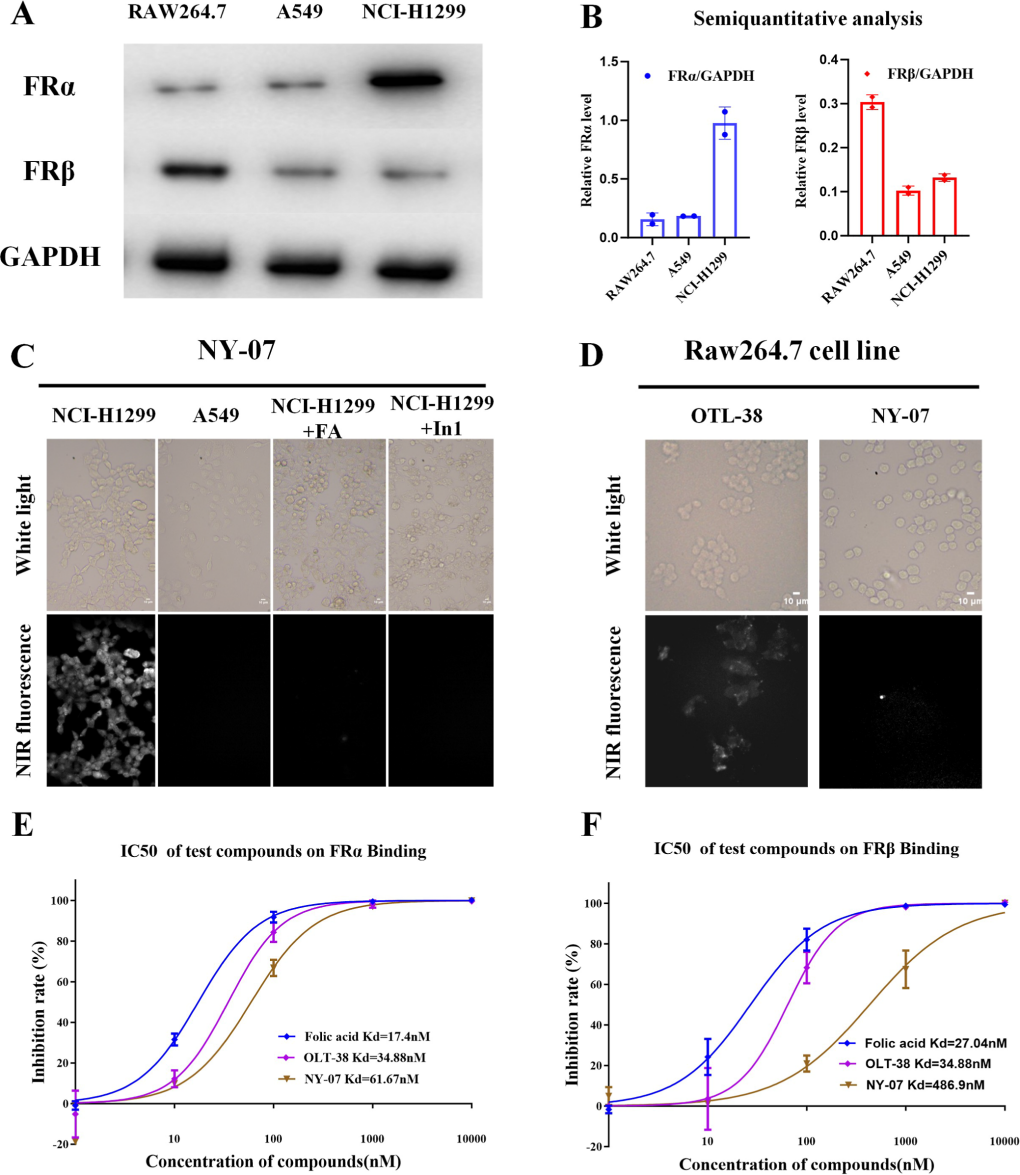
Targeted NIR fluorophores NY-07 and OTL-38 during cell-based assays. (A) Expression of FRα and FRβ protein in NCI-H1299, A549, and RAW264.7 cells and corresponding quantitative analysis of bonds, and GAPDH was used for normalization. (B) Semiquantitative analysis of FRα and FRβ. The Western blot band intensities were measured with ImageJ software. (C) Live-cell binding assay for NY-07 in FRα^+^ NCI-H1299 and FRα^–^ A549 melanoma cell lines and RAW264.7 (FRβ^+^) after incubation with 100 nM of each molecule for 1 h. Block group was pro-incubated with folic acid, and inhibitor 1­(NaN_3_^+^ deoxyglucose) inhibits the endocytic process that consumes ATP. (D) Live-cell binding assay for NY-07 and OTL-38 in FRβ^+^ RAW264.7 after incubation with 100 nM of each molecule for 1 h at 37 °C. Scale bar = 10 μm. (E) Assessment of *K*_d_ of folate, OTL38, and NY-07 to FRα. Error bars represent SD (*n* = 3). (F) Assessment of *K*_d_ of folate, OTL38, and NY-07 to FRβ. Error bars represents SD (*n* = 3).

### Evaluation of In Vivo Efficacy and Specificity of NY-07

Whole-body imaging of nude mice bearing FRα^+^ NCI-H1299 tumor xenografts showed strong tumor uptake compared to nude mice bearing FRα^–^ A549 tumor xenografts, consistent with FRα expression results (Figure 3A,B). Competitive inhibition results showed that NCI-H1299 no longer took up NY-07 in the blocked group, while the unblocked group exhibited high fluorescence intensity 24 h later (Figure 3C,D). SKOV-3 and MDA-MB-231 tumor-bearing mouse model imaging results indicated that FRα overexpression ovarian cancer and triple-negative breast cancer showed strong fluorescence at the tumor location (Figure S11). Ex vivo imaging of all major organs and tissues confirmed that tissue retention was limited to organs where FRα is known to be of overexpression, specifically in the kidneys and tumors (Figure 3E,F). In the competitive inhibition experiment, the tumor fluorescence intensity of the blocked group was significantly lower than that of the unblocked group, but more NY-07 accumulated in the kidneys (Figure 3G). Excluding FRα^+^ kidney, all tumor/tissue ratios were sufficient to achieve good tumor contrast, with TBR ranging from 2.6:1 (tumor/liver), 5.8:1 (tumor/muscle), 5:1 (tumor/lung), 5.6:1 (tumor/heart), and 4:1 (tumor/intestine) to 5.45:1 (tumor/spleen) (Figure S12). Histologic analysis showed that NY-07 was able to image single tumor cells in NCI-H1299 tumors, while no fluorescence was detected in A549 tumors. Immunohistochemical staining (IHC) analysis of FRα and FRβ on NCI-H1299 and A549 tumors revealed that FRα was expressed in NCI-H1299 tumors, correlating with the observed NIR fluorescence signal (Figure 3H).

**Figure 3:**
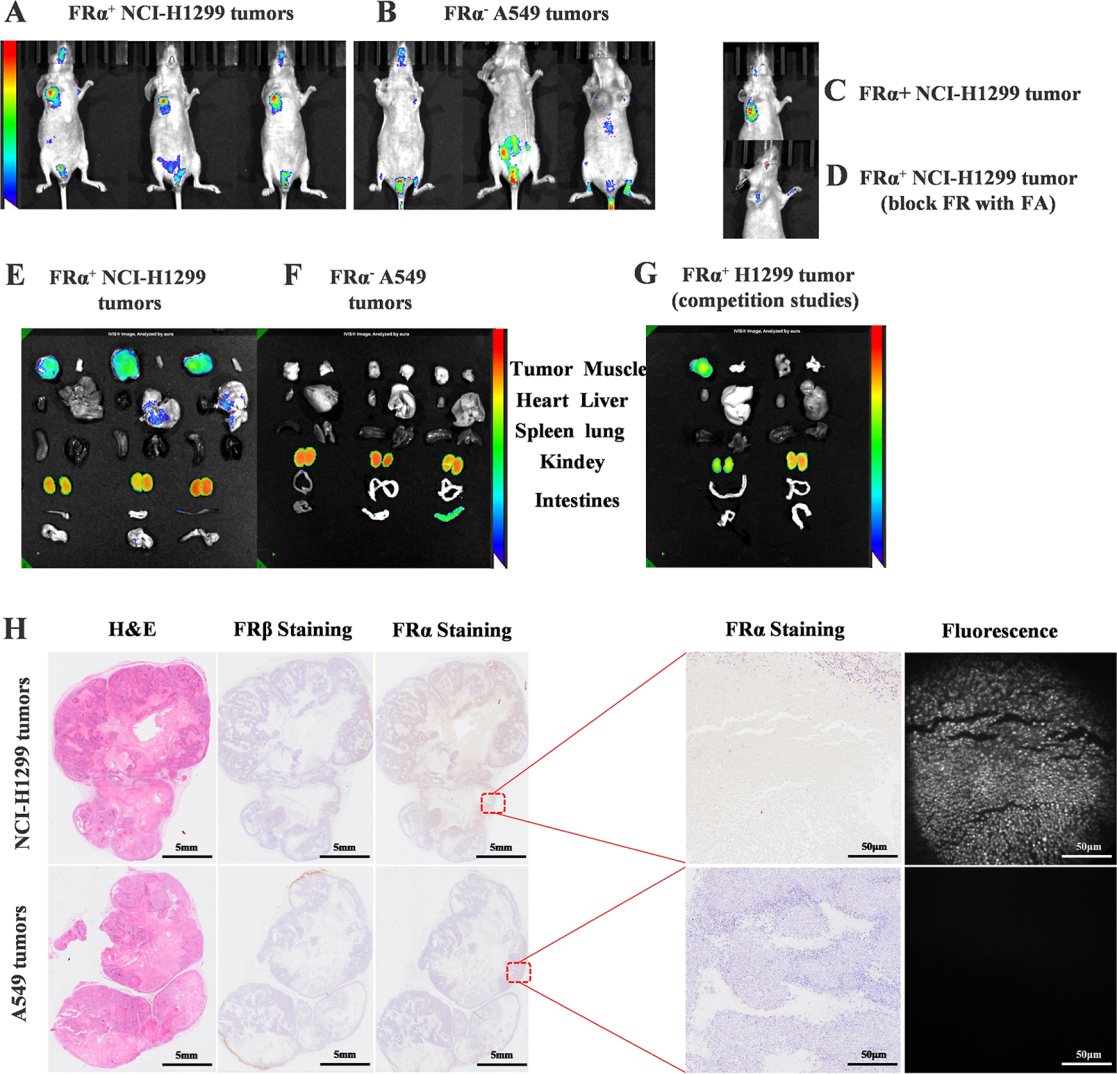
In vivo efficacy and specificity of NY-07. Representative IVIS images showing overlay of fluorescence images over white light images of mice bearing. (A) FRα^+^ NCI-H1299 tumors, (B) FRα^–^ A549 tumors, (C) FRα^+^ NCI-H1299 tumors (half body), and (D) FRα^+^ NCI-H1299 tumors (competition with 100-fold excess FA) 24 h after administering 10 nmol of NY-07. Ex vivo tissue biodistribution of NY-07 in mice with (E) FRα^+^ NCI-H1299 and (F) FRα^–^ A549 tumors 24 h after administering 10 nmol of NY-07 using IVIS imaging system (*n* = 3). (G) Ex vivo tissue biodistribution from the competition (in the absence (left) and presence (right) of 100-fold excess of FA) studies. (H) Whole-section images were obtained and evaluated using H&E staining, FRα IHC, FRβ IHC, and NIR microscopy Scale bar = 5 mm and 50 μm.

### Distinguishing Cancerous and Inflammatory Tissues

It is not an unusual occurrence for cancer patients to experience concomitant inflammatory or autoimmune disorders, including but not limited to fibrosis, arthritis, pneumonia, ulcerative colitis, or pathogenic infections.^28^ In particular, the fact that tumors and inflammation manifest in the same organ greatly increases the complexity of diagnosis and treatment.^29^ Surgeons can sometimes distinguish between tumors and inflammation during surgery based on their visual appearance and other characteristics such as texture and consistency. However, the inability of clinicians to distinguish between tumors and inflammation during surgery can have several potential dangers, including recurrence due to incomplete tumor removal and damage to surrounding healthy tissues.^26,30^ Currently the only FDA-approved probes are ICG and OTL-38. In a study of ICG as a fluorescence imaging of breast cancer tumors, the false positive rate was as high as 68.3%.^31^ In another phase III clinical trial of OTL-38 for ovarian cancer, there was a false positive rate of 24.8%.^32^ We innovatively used pemetrexed as the targeting moiety to synthesize NY-07 and detected that the *K*_d_ value of NY-07 for FRα was 8-fold higher than that for FRβ. This specificity was confirmed by subsequent cell-targeting experiments, and NY-07 showed specificity only for the FRα-overexpression cell line, whereas OTL-38 could still detect fluorescence in macrophages due to its similar affinity for FRα and FRβ receptors. In vivo imaging experiments confirmed that the imaging mechanism of NY-07 is achieved by FRα uptake, which can accurately identify tumor boundaries and mitigate the risk of false positives caused by FRβ uptake in inflamed tissues, thereby reducing unnecessary healthy tissue resection and facilitating patients’ postoperative recovery. NY-07 has currently obtained IND approval from NMPA and FDA, and it has demonstrated excellent biosafety in Phase 1 clinical trials. These results indicate the potential advantages of NY-07 for future clinical applications.

An inflammation model was induced surrounding tumors via lipopolysaccharide (LPS) injection, followed by administration of 10 nmol of NY-07 and OTL-38 for imaging 96 h later. The NY-07 group showed high contrast between the tumor and the surrounding inflammatory area, while the OTL-38 group showed high fluorescence intensity in both locations (Figure 4A). IHC confirmed FRβ overexpression in the inflamed areas. The OTL-38 group showed high fluorescence in the inflammatory area, while no fluorescence was detected in the NY-07 group using fluorescence microscopy (Figure 4B,C). The average fluorescence intensity for NY-07 group tumors was measured as follows: (3.57 ± 0.76) (photons/sec/cm^2^/sr) × 10^8^ and inflammatory area (1.36 ± 0.17) (photons/sec/cm^2^/sr) 10^8^ (**p < 0.01, Student’s *t*-test) (Figure 4D). The average fluorescence intensity for the tumors in the OTL-38 group was measured as follows: (3.88 ± 1.88) (photons/sec/cm^2^/sr) × 10^8^ and inflammatory area (2.45 ± 0.81) (photons/sec/cm^2^/sr) × 10^8^ (*p* = 0.3, Student’s *t*-test) (Figure 4E). The tumor to inflammation values for the NY-07 group and OTL-38 group were determined as 2.61 ± 0.23 and 1.51 ± 0.34, respectively (**p < 0.01, Student’s *t*-test) (Figure 4F). The results strongly indicated that NY-07 exhibits in vivo targeting capabilities for FRα, enabling efficient discrimination of tumor and surrounding FRβ overexpression inflammatory tissues.

**Figure 4:**
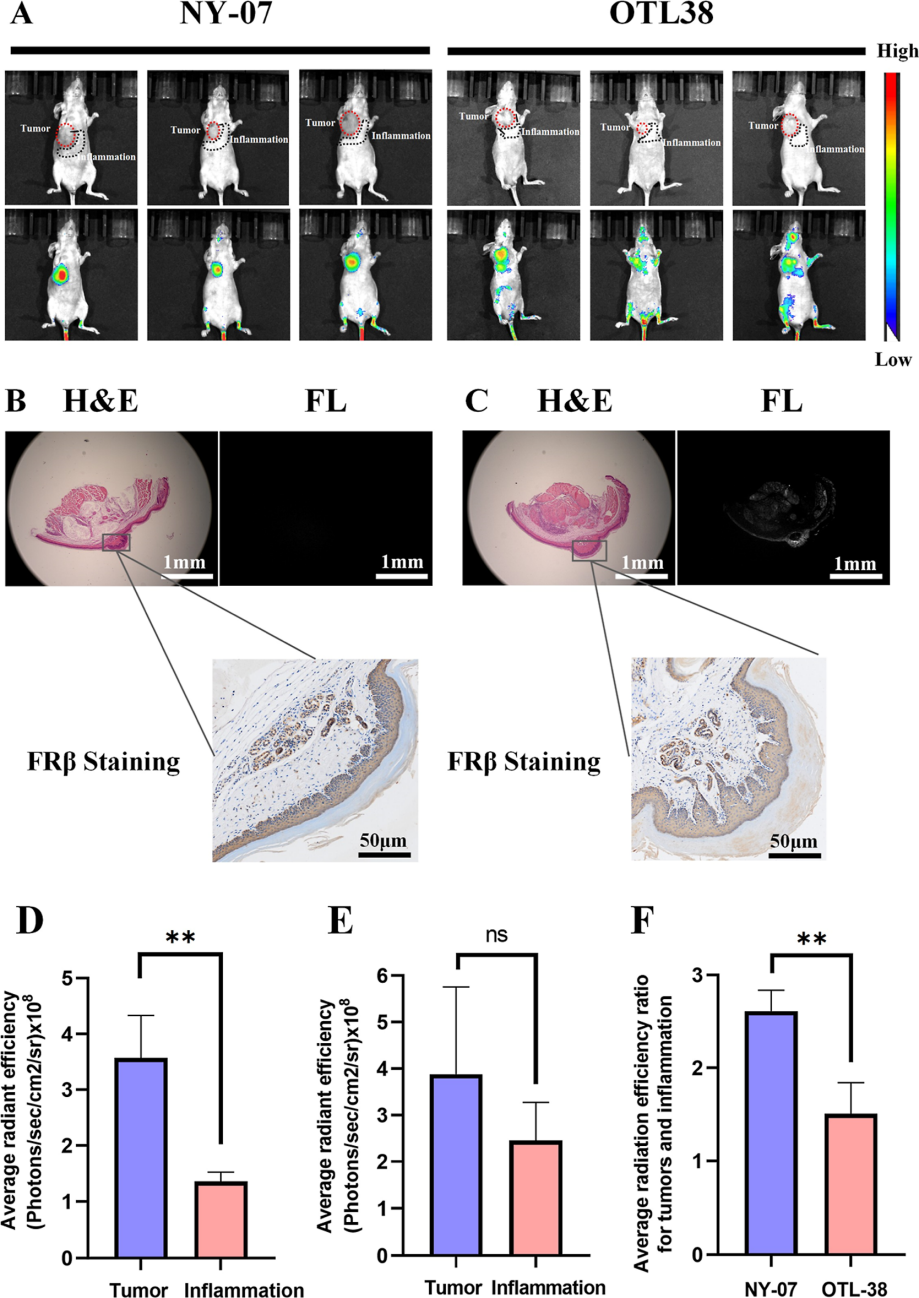
Comparing NY-07 and OTL-38 for differentiating tumor and inflammation imaging. (A) Analysis of NY-07 and OTL-38 accumulation in mice with peritonitis treated with LPS (*n* = 3), taken at 96 h after tail vein injection of NY-07 and OTL-38. (B) Whole-section images were obtained using H&E staining, FRβ IHC, and NIR microscopy of the NY-07 group. (C) Whole-section images were obtained using H&E staining, FRβ IHC and NIR microscopy of the OTL-38 group. (D) Average radiant efficiency of NY-07 in tumors and in inflammation areas (*n* = 3) (ns: no significant difference, Student’s *t*-test). (E) Average radiant efficiency of OTL-38 in tumors and in inflammation areas (*n* = 3) (**p < 0.01, Student’s *t*-test). (F) Average radiant efficiency ratio for tumors to inflammation in the NY-07 and OTL-38 group (*n* = 3) (**p < 0.01, Student’s *t*-test) scale bar = 1 mm and 50 μm.

### Molecular Imaging of Carrageenan-Induced Inflammation

In order to verify that NY-07 cannot specifically bind to inflammation, we established a carrageenan-induced inflammation model. The experimental flowchart is shown in Figure 5A. In the carrageenan-induced inflammation model, fluorescence signals could be clearly observed at the inflammatory site of the toe after injection of OTL-38 (*n* = 3), but the NY-07 group (*n* = 3) showed no significant difference in the fluorescence signal at the inflammatory site after administration (Figure 5B). Compared with OTL-38, the fluorescence intensity of the inflammatory site in the NY-07 group was significantly lower than that of OTL-38 at each time point (****p < 0.0001, Student’s *t*-test) (Figure 5C). The ratio of the fluorescence intensity of the right foot to the fluorescence intensity of the left foot remained at around 2.5 after 1 h, and reached the maximum value 3.38 ± 0.84 at 24 h. This result demonstrated the binding specificity of OTL-38 to inflammation. The ratio of the fluorescence intensity of the right foot to the fluorescence intensity of the left foot remained at around 1.2 at any time point, reaching the maximum value 1.48 ± 0.71 at 8 h. The ratio of OTL-38 significantly higher than NY-07 group (****p < 0.0001, Student’s *t*-test) (Figure 5D). The results confirmed that NY-07 could not specifically bind to inflammation, effectively avoiding false positive phenomenon caused by inflammation. H&E sections confirmed that there was a large amount of lymphocyte infiltration and synovial hyperplasia in the inflammatory site (Figure 5E).

**Figure 5:**
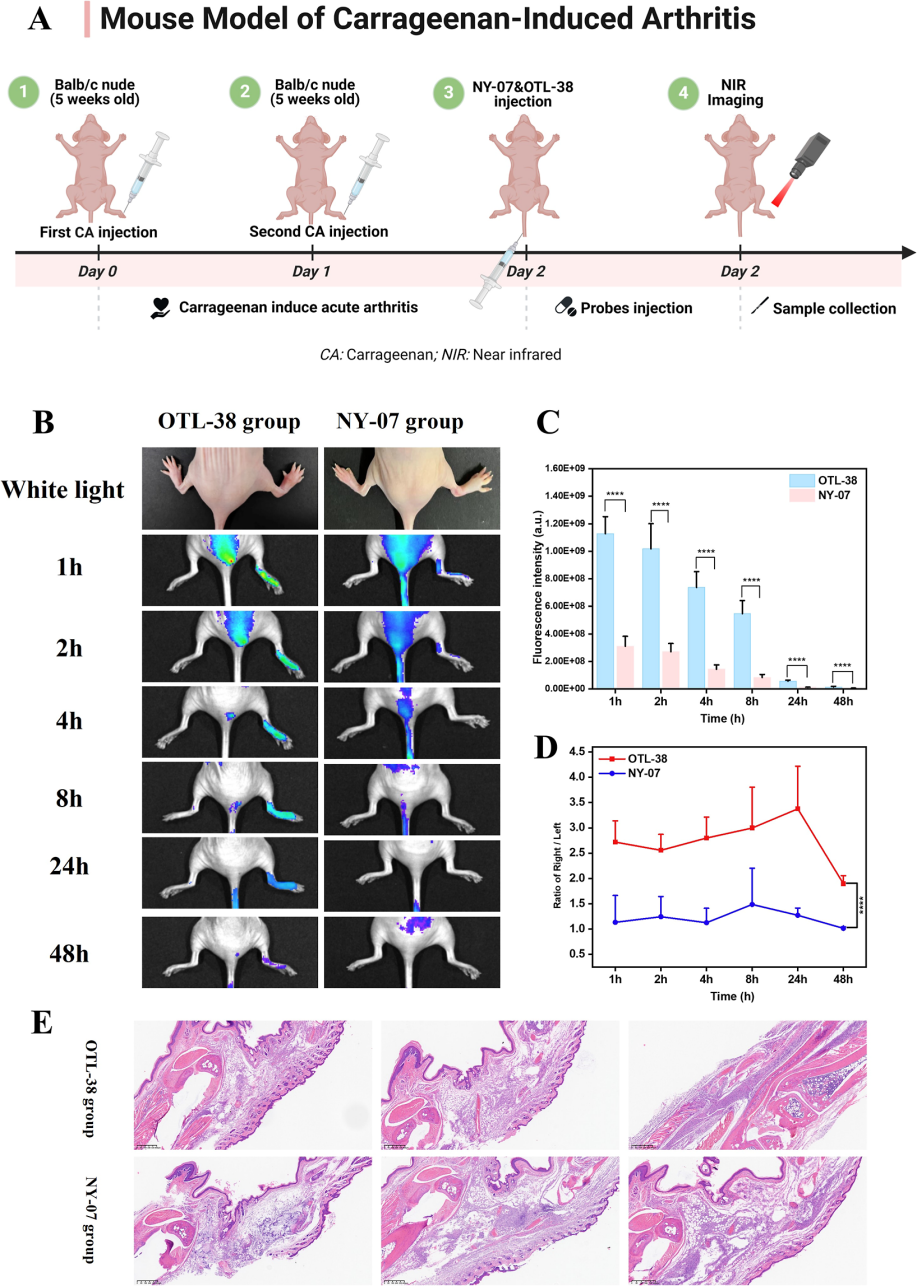
Application of near-infrared in vivo imaging of NY-07 and OTL-38 in the carrageenan-induced arthritis model. (A) Experimental flowchart. (B) NY-07 and OTL-38 were imaged using the IVIS imaging system at 1, 2, 4, 8, 24, and 48 h (*n* = 3). The right foot is arthritic, and the left foot is normal. (C) Fluorescence intensity change trend of the right foot in NY-07 and OTL-38 groups (*n* = 3) (****p < 0.0001) (Student’s *t*-test). (D) The ratio of the fluorescence intensity of the right foot to the fluorescence intensity of the left foot (*n* = 3) (****p < 0.0001) (Student’s *t*-test). (E) H&E sections of arthritis models. Scale bar = 400 μm.

### Distinguishing Pneumonia from Normal Lung Tissues

To better characterize the patterns of NY-07, OTL38, and ICG accumulation within inflammation and confirm accumulation in FRβ^+^ inflammation areas, we built acute lung injury and showed experimental flowchart in detail (Figure 6A). Acute lung injury was induced via intranasal administration of lipopolysaccharide (LPS) with validation of the model conducted through Western blot (WB) (Figure 6B,C). Following this, 10 nmol NY-07, OTL-38, and indocyanine green (ICG) were administered intravenously. The mice were anesthetized, and the lung tissues were removed. The excised lungs were subjected to H&E staining and IHC. All sections underwent microscopic tumor mapping using NIR fluorescence microscopy and molecular correlative analysis using a combination of H&E staining and FRα FRβ IHC. IHC analysis showed brown FRβ-positive areas in all lung tissues, and NIR fluorescence signals were detected in FRβ-positive sites for both the OTL-38 and ICG groups but were absent in the NY-07 group under fluorescence microscopy (Figure 6D). This outcome demonstrates that NY-07 exhibits low affinity for FRβ, despites its high macrophage-associated expression in inflammatory regions, thereby effectively avoiding false-positive signals in inflamed tissues.

**Figure 6:**
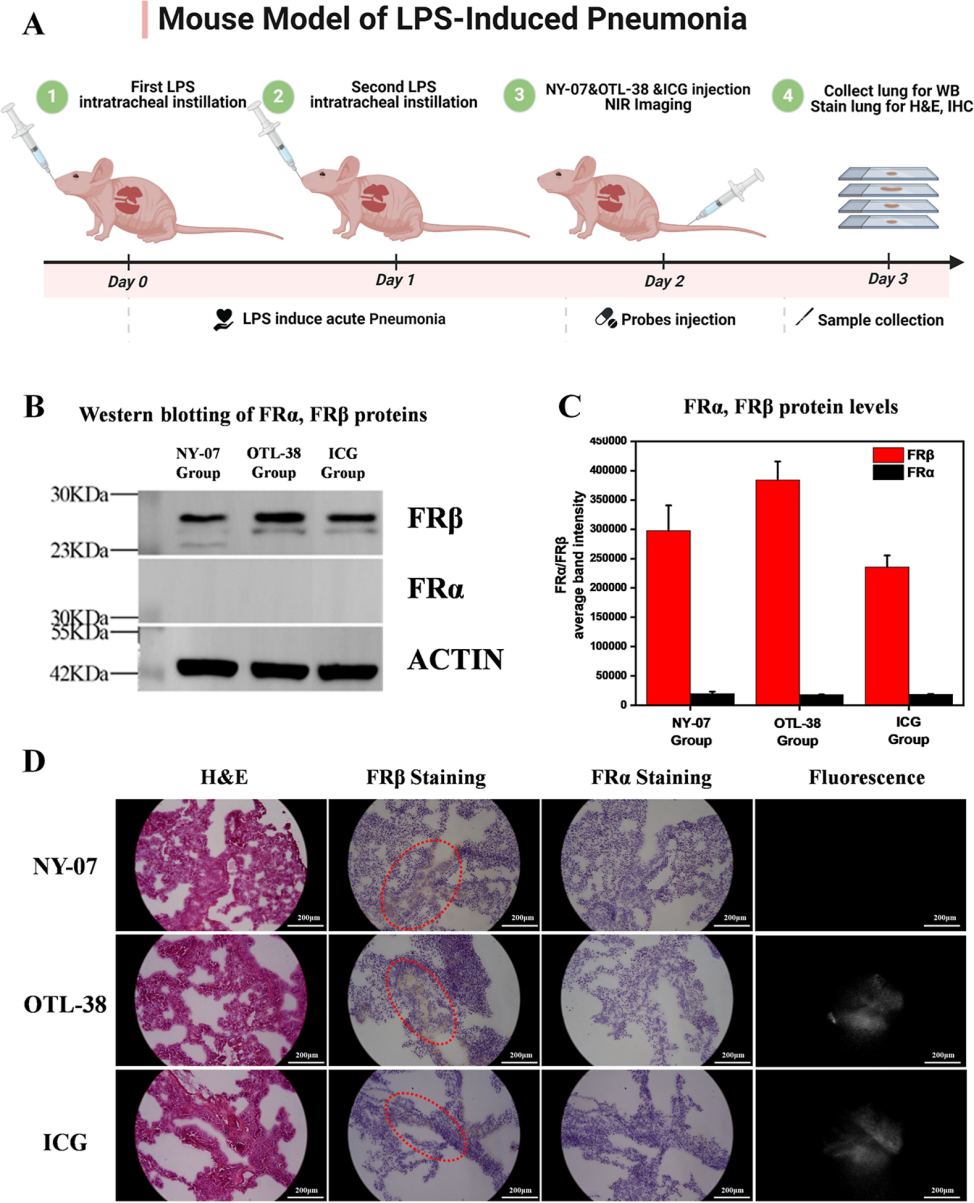
Comparison of NY-07, OTL-38, and ICG in distinguishing pneumonia from normal lung tissues. (A) Experimental flowchart. (B) Expression of FRα and FRβ protein in lung tissues. (C) Quantitative analysis of FRα and FRβ protein. (D) Whole-section images were obtained using H&E staining, FRβ IHC, and NIR microscopy for NY-07, OTL-38, and ICG group. Scale bar = 200 μm.

### Determination of NY-07 Tumor-to-Background Ratios

Ideal tumor-targeting probes should exhibit high accumulation at tumors while being rapidly cleared from normal tissues. Pemetrexed has a high affinity for the FRα, and the α-carboxylic acid undergoes polyglutamylation by folyl-polyglutamate synthetase (FPGS), prolonging its intracellular action time.^33^ NY-07 introduces fluorescent motif S0456 into the tail of pemetrexed while retaining the α-carboxylic acid at the tail, which still entered the cell via endocytosis via FRα. The larger molecular structure inevitably prolongs its imaging time inside the cell.^34^ This is confirmed by our animal imaging results. At various time points after NY-07 injection, the imaging results showed that the axillary tumor had a great contrast compared with the surrounding tissue (Figure S13A). Although the fluorescence intensity of both the tumor and background areas decayed over time, the background fluorescence intensity decayed faster than that of the tumor (Figure S13B). The tumor fluorescence intensity 24 h after administration decreased by about 75% compared with the first hour after administration, while the background fluorescence intensity decreased by 82%. The tumor to background ratio trend showed that the clinically required TBR (TBR >1.5) can be achieved from 1 h after injection and the highest TBR = 3.23 ± 0.28 reached at 6 days. NY-07 had a longer retention time in tumor tissue than in normal tissue, and its excellent fluorescence properties allowed it to show excellent contrast at lower dose. As shown in Figure S13C, TBR can still be maintained at 2.75 ± 0.37 after 12 days, thus providing a longer surgical window. The rapid clearance indicated a low biological toxicity and high biosafety (Figure S14). The persistence of NY-07, OTL-38, and ICG tumor imaging were compared at the same dose of 10 nmol/per. The results showed that NY-O7 and OTL-38 could still detect strong fluorescence in the tumor after 12 days, whereas ICG was almost completely cleared from the body on the sixth day. The rapid clearance indicated low biological toxicity and high biosafety (Figure S15).

### Submillimeter Hepatocellular Carcinoma Imaging Using NY-07

The liver is also a common site of lung cancer metastasis, and about 28–33% of lung cancer cases have liver metastases.^35^ Ultrasmall tumors seriously affect the postoperative prognosis and survival of patients. However, the lack of effective intraoperative diagnosis is one of the key factors leading to residual tumor cells and postoperative recurrence. The NIRF imaging system have been widely used for tumor localization during liver cancer surgery.^36,37^ In this study, we used the NIRF imaging system to detect three tiny bright spots about 1 mm in diameter and removed them under the guidance of the real-time NIRF imaging system. 24 h after intravenous injection of 10 nmol NY-07, the liver of BALB/c nude mice was imaged by FLI-10B after laparotomy. Three lesions were clearly observed on the liver surface, each of which was approximately 1 mm in size. These spots were also be clearly identifiable on the ex vivo organ (Figure S16A). H&E section scan confirmed that these tiny fluorescent spots corresponded to early liver cancer lesions. The diameter for the 2 tumors was measured as follows: tumor 1: long diameter 653 μm and short diameter 1156.9 μm, and tumor 2: long diameter 765.1 μm and short diameter 552.1 μm (Figure S16B). ImageJ was used to measure the fluorescence intensity of the three carcinoma spots and each ex vivo organ. The results showed that the ratio of tumor average fluorescence intensity to each ex vivo organ was measured as follows: tumor to muscle = 11.66 ± 1.73, tumor to liver = 2.31 ± 0.34, tumor to heart = 9.23 ± 1.37, tumor to spleen = 7.23 ± 1.07, tumor to colon = 5.34 ± 0.79, tumor to intestine = 8.03 ± 1.19, and tumor to spleen = 0.97 ± 0.14, indicating that NY-07 can effectively locate submillimeter tumor lesions (Figure S16C).

### Fluorescence Imaging of NY-07 in the Orthotopic Colorectal Mice Model

Colorectal cancer has become the second most prevalent cancer type in China 2024.^38^ Previous studies show that FRα positivity rates are 44% in metastatic colorectal cancer tissues and 33% in primary tumors, indicating that FRα is an attractive target for colorectal cancer diagnosis and treatment.^39,40^ Although colorectal tumor surgery is now often performed by colectomy, where achieving R0 resection remains challenging due to the lack of intraoperative real-time tumor margin imaging by surgeons.^34,41,42^ We used AOM/DSS to induce related colorectal cancer and evaluated the application of the NY-07 probe for in situ colorectal cancer imaging. FLI-10B was used to detect the areas of high fluorescence in isolated colorectal segments (Figure S17A). H&E sections confirmed the cancerous areas, which showed high fluorescence signals under a fluorescence microscope (Figure S17B). Subsequently, a 3D simulation of the fluorescence distribution across different intestinal segments was performed (Figure S17C), and ImageJ was used to quantitatively detect the fluorescence intensity of CRC and normal colorectal tissue (Figure S17D). Finally, we analyzed the NY-07 imaging tumors in 3 mice, and the result showed fluorescence intensity of tumors were significantly higher than surrounding normal intestinal tissue (94.32 ± 12.52 vs 39.39 ± 3.87, ***p < 0.0001, Student’s *t*-test) (Figure S17E). These results effectively confirm the potential of NY-07 to accurately locate tumor boundaries during colorectal tumor surgery.

## Conclusions

In summary, we developed NY-07, a pemetrexed-derived fluorescent probe, that selectively targets folate receptor α (FRα) with a dissociation constant (*K*_d_) of 61.67 nM compared to 486.9 nM for FRβ. In vivo, the probe achieved tumor-to-background ratios above 3, meeting clinical imaging thresholds, enabled detection of lesions smaller than 1 mm across multiple tumor types and demonstrated favorable biosafety, photostability, and a prolonged imaging window. Histological analysis combined with fluorescence microscopy further confirmed FRα-specific localization at the cellular level. By establishing selective FRα targeting, NY-07 addresses a long-standing source of false positives in fluorescence-guided surgery and provides a concrete foundation for clinical translation, reducing errors from FRβ-positive inflammatory macrophages and enabling precise tumor margin delineation. Extending this strategy into the NIR-II window, where deeper tissue penetration can be achieved, represents the next step, and future probe designs will benefit from combining NY-07’s molecular selectivity with optimized optical properties. To realize this potential, new approaches will be needed to overcome the poor solubility and limited specificity that have hindered the development of current small-molecule NIR-II probes.

## Supplementary Material



## Data Availability

The main data supporting the results of this study are available within the paper and its Supporting Information. The raw near-infrared fluorescence images can be obtained after asking the corresponding authors and clarifying the purpose of use.

## Notes

Fluorescence performance test of NY-07, metabolism experiment, organ distribution and biosafety of NY-07, synthesis methods, materials and instrumentation, structural characterization, ^1^HNMR Spectra, HPLC Spectra and MS analysis of NY-07 (PDF)
