# Supplementary material for: Fluorescence Discrimination of Cancer from Inflammation by Selective Targeting Folate Receptor α
Source: Chem Biomed Imaging. 2025 Oct 2;4(3):326–38. doi: 10.1021/cbmi.5c00087 (PMC13014312; doi:10.1021/cbmi.5c00087)
Supplement: Supplementary file 1 [file im5c00087_si_001.pdf]

SUPPORTING INFORMATION

Fluorescence Discrimination of Cancer from Inflammation by Selective Targeting Folate Receptor α

Yunlong Li[1†], Nida El Islem Guissi[1†], Junming Dong[1], Sunil Singhal[2], Bo Dai[3*], Christopher Butch[1*], Huiming Cai[4*], Yiqing Wang[1*]

[1]Department of Biomedical Engineering, College of Engineering and Applied Sciences, State Key Laboratory of Analytical Chemistry for Life Science, Nanjing University, Nanjing 210023, China;

[2]University of Pennsylvania, Departments of Radiation Oncology, Medicine and Surgery, Philadelphia, PA 19104, USA

[3]Department of Thoracic Surgery, Nanjing Drum Tower Hospital, The Affiliated Hospital of Nanjing University Medical School, Nanjing 210008, China;

[4]Nanjing Nuoyuan Medical Devices Co. Ltd, Nanjing 211500, China.

*Corresponding authors: wangyiqing@nju.edu.cn, caihuiming@nuoyuanmedical.cn, chrisbutch@nju.edu.cn, 1039610132@qq.com.

† These authors contributed equally to this work

Table of Contents

Experimental Procedures 2
Materials and Instrumentation 2
Synthesis and characterization 3
^{1}HNMR Spectra 6
Fig. S1. ^{1}HNMR spectrum of 2 in DMSO-*d6*. 6
Fig. S2. ^{1}HNMR spectrum of 3 in DMSO-*d6*. 7
Fig. S3. ^{1}HNMR spectrum of 4 in DMSO-*d6*. 7
Fig. S4. ^{1}HNMR spectrum of 7 in DMSO-*d6*. 8
Fig. S5. ^{1}HNMR spectrum of NY-07 in DMSO-*d6*. 8
HPLC Spectra of NY-07 9
MS analysis of NY-07 9
Summary of Fluorescence performance 10
Compare the stability of NY-07 and OTL-38 in PBS solution 11
Docking studies of NY-07 OTL-38 Pemetrexed and Folic 12
In vivo efficacy and specificity of NY-07 13
Quantitative analysis of fluorescence intensity of isolated organs 14
NY-07 accumulates in FRα$^+$ NCI-H1299 Xenografts 15
Toxicity of NY-07 & folic acid (FA) toward L02 cell line 16
Long-term imaging of NY-07, OTL-38, ICG 16
Localization of NY-07 in liver microtumors 17
In vivo efficacy and specificity of probe NY-07 in colorectal cancer mice model 18

Experimental Procedures

Materials and Instrumentation

All other chemicals of analytical grade were acquired from Aladdin, Sigma-Aldrich, Fisher, and Alfa Aesar and used as received unless otherwise indicated.

Nuclear magnetic resonance (NMR) data including ^{1}H were obtained using a Bruker Avance III 600 MHz NMR spectrometer, and chemical shifts as δ were reported in ppm with tetramethylsilane as the internal standard. Analytical high-performance liquid chromatography (HPLC) with a diode array detector was performed with an Agilent 1100 System. For liquid chromatography-mass spectrometry (LC-MS), a Waters 2695 Alliance HPLC System coupled with a Waters 2998 diode array detector and a Waters 3100, SQ mass spectrometer was employed. An Agilent XDB C18 column, 3.5 μm particle size, in a 3 mm by 100 mm column or a Waters XBridge C18 3.5 μm, 3 mm by 50 mm were used for both LC techniques. For diode array analysis, wavelengths from 200-900 nm were collected in the Agilent HPLC and 200-800

nm in the Waters LC-MS. Mass spectral data were collected in both positive and negative modes on a Waters 3100 SQ Mass Spectrometer.Confocal imaging was taken with Leica SP8 STED 3X.

Synthesis and characterization

2,3,3-Trimethyl-3*H*-indole-5-sulfonic acid (2).

A mixture of 4-hydrazinobenzenesulfonic acid (20 g, 97.5 mmol), sodium acetate (16 g, 195 mmol), and 3-methyl-2-butanone (14.9 mL, 139 mmol) in glacial acetic acid (97 mL) was heated at 110°C under a nitrogen atmosphere in a sealed tube. The crude product was filtered, washed with methyl *tert*-butyl ether (MTBE), and collected after precipitation as a brown solid (19.4 g, 83%); mp 292 to 293°C; 1H NMR (400 MHz, DMSO-*d6*): δ 1.25 (s, 6*H)*, 2.22 (s, 3*H)*, 7.10 to 7.30 (br. s, 1*H)*, 7.36 (d, *J* = 8.0 Hz, 1*H)*, 7.58 (d, *J* = 8.0 Hz, 1*H)*, 7.65 (s, 1*H)*. 13C NMR (100 MHz, DMSO-*d6*): δ 15.0, 22.4, 53.1, 118.5, 125.0, 145.0, 153.5, 171.5, 173.4, 188.8.

Potassium 2,3,3-Trimethyl-3H-indole-5-sulfonate (2K).

The obtained **2** (65 g, 254 mmol) was dissolved in methanol (500 mL). Potassium hydroxide solution prepared from potassium hydroxide (17 g, 300 mmol) and 2-propanol (200 mL) in a gentle heating condition was added into **2** solution using a dropping funnel for 1 h. The crude mixture was filtered, washed with 2-propanol and ethyl acetate, and collected as a brown solid (55 g, 78%); mp 292 to 293°C; ^{1}HNMR (400 MHz, DMSO-*d6*): δ 1.254 (s, 6*H*), 2.22 (s, 3*H*), 7.10-7.30 (br. s, 1*H*), 7.36 (d, *J* = 8.0 Hz, 1*H*), 7.58 (d, *J* = 8.0 Hz, 1*H*), 7.65 (s, 1*H*). ^{13}C NMR (400 MHz, DMSO-*d6*): δ 15.0, 22.4, 53.1, 118.5, 125.0, 145.0, 153.5, 171.5, 173.4, 188.8. HRMS (ESI) calculated for C11H13NO3S *m/z* 239.06, found [M+H]+ *m/z* 240.08.

5-Sulfo-1-(4-sulfobutyl)-2,3,3-trimethyl-3*H*-indolium betaine (3)

2K (1.00 g, 4.18 mmol, 1.0 eq.) and 1,4-butanesultone (1.00 mL, 1.33 g, 9.78 mmol, 2.3 eq.) were suspended in 1,2-dichlorobenzene (25 mL) and stirred at 110 °C for 12 hours under argon. After cooling to room temperature, H_2O was added to the reaction mixture until all solid material was dissolved. The aqueous phase was washed with CH2Cl2 and the organic layer was discarded. After lyophilization, **product** was obtained as red solid (1.04 g, 2.80 mmol, 66%). **1H NMR** (600.13 MHz, D2O): *δ* = 1.60 (s, 6H, H-10/11), 1.76 - 1.79 (m, 2H, H-15), 1.85 - 1.94 (m, 2H, H-14), 2.97 (t, 2H, 3*J*H-H = 7.45 Hz, H-16), 4.12 (t, 3*J*H-H = 6.00 Hz, 2H, H-13), 7.91 (d, 3*J*H-H = 8.60 Hz, 1H, H-6), 8.02 (d, 3*J*H-H = 8.60 Hz, 1H, H-7), 8.11 (s, 1H, H-4); the signal for H-12 was not observed; **MS** (HR-ESI-): m/z = 374.0719 [M-H]-, calculated for C15H20NO6S2-: 374.0738.

2-((E)-2-((E)-2-chloro-3-(2-((E)-3,3-dimethyl-5-sulfonato-1-(4-sulfonatobutyl)indolin-2-ylidene)ethylidene)cyclohex-1-en-1-yl)vinyl)-3,3-di

methyl-1-(4-sulfonatobutyl)-3H-indol-1-ium-5-sulfonate(4:S0456)

The product 3 (2.58 g, 6.89 mmol) was dissolved in 4 mL water at 30 °C. After the addition of sodium acetate (1.35 g, 13.8 mmol), the mixture was diluted with 15 mL isopropyl alcohol. (3) (1.11 g, 3.45 mmol), was added, followed by addition of acetic anhydride (1.39 g, 13.8 mmol). The reaction mixture was heated to reflux and stirred for 30 min after which it was cooled to room temperature. The product was collected by vacuum filtration and washed with two rinses of isopropyl alcohol. The damp solid was triturated twice with 20 mL of refluxing methanol, filtered, and dried under vacuum to yield the chloro dye product (4) (5.43 g, 6.55 mmol, 95.0% yield). 1H NMR (500 MHz, D2O) δ 8.12 (d, J = 13.8 Hz, 2H), 7.82-7.73 (m, 2H), 7.69-7.62 (m, 2H), 7.19 (d, J = 8.4 Hz, 2H), 6.06 (d, J = 14.0 Hz, 2H), 4.03 (s, 4H), 2.87 (t, J = 7.4 Hz, 4H), 2.35 (s, 4H), 1.91-1.72 (m, 8H), 1.57 (s, 14H). LC-MS (ES-), calculated for C38H48ClN2O12S4: 886.17008; LC-MS,found [m/z]-: 885.56 (M-H), 442.49 (M-2H/2); high resolution mass spectrometry (HRMS), found [m/z]-:885.1608, [M-H]-, 442.0762 (M-2H/2), 294.38207 (M-3H/3); UV-Vis λmax= 783.53 from LC-MS DAD in methanol/buffer. Analytically pure samples of chloro dye (4) were obtained by preparative chromatography on a C-18 column using a 10-50% acetonitrile–0.02 M trifluoroacetic acid buffer system. The sample was loaded in 0.1 M trifluoroacetic acid. Typically 250 mg of crude dye was chromatographed followed by assaying dye-containing fractions by LC-MS, combining, concentrating, and lyophilizing pure fractions three times, followed by ion exchange to the sodium salt and lyophilization to give the final product, chloro dye .

Tert-butyl2-(4-(2-(2-amino-4-oxo-4,7-dihydro-1H-pyrrolo[2,3-d]pyrimidin-5-yl)ethyl)benzamido)-3-(4-(tert-butoxy)phenyl)propanoate(6)

To a solution of Pemetrexed hydrolyzed acid (0.2g, 0.67 mmol, 1 equiv), HATU (0.305 mg, 0.804 mmol, 1.2 equiv), and NH_2-L-Tyr(OtBu)-OtBu.HCl (0.265 g, 0.804mmol, 1.2 equiv) in anhydrous DMF (147 mL) at 23°C under argon, DIPEA (0.346 g, 2.68 mmol, 4.0 equiv) was added slowly over a period of 10 min. The reaction mixture was stirred at 23°C under argon for 2 h. The reaction mixture was cannulated as a steady stream to a stirred solution of 0.1 N aq. HCl (500 mL, 0.07 M) over the period of 30 min to give blue precipitate of compound. The precipitate was filtered using sintered funnel under aspirator vacuum and washed with water (2×300 mL) until the pH of thefiltrate was between 3 and 4. The wet solid was allowed to dry under high vacuum for 12 h to obtain compound2(16.24 g, 96.7%).1H-NMR (500 MHz, DMSO-d6/D2O)δ8.65 (s, 1H, Pyr−CH), 7.81−7.77 (m, 2H, Pte−Ar−CH), 7.59 (d,J= 8.1 Hz, 2H, Pte−Ar−CH), 7.19−7.13 (m, 2H, Tyr−Ar−CH), 6.88−6.81 (m, 2H, Tyr−Ar−CH), 5.12 (s, 2H, Pte-CH2), 4.49 (dd,J= 8.9, 6.8 Hz, 1H, Tyr-αCH), 3.01 (t,J= 7.4 Hz, 2H, Tyr-CH2), 1.30 (s, 9H,tBu-CH3), 1.20(s, 9H,tBu-CH3).13C-NMR (125 MHz, DMSO-d6/D2O)δ170.88,165.66, 160.13, 155.92, 155.63, 153.61, 153.38, 149.19, 145.37,141.67, 134.48, 132.26, 130.11, 129.76, 128.78, 128.47, 128.20,123.68, 117.34, 115.03, 80.81, 77.82, 54.98, 53.87, 40.09, 40.00, 39.93, 39.83, 39.76, 39.67, 39.50, 39.33, 39.17, 39.00, 35.97, 28.57, 27.66. HRMS (ESI) calcd for C33H36F3N7O6[M + H]+ m/z 573.6;found:m/z 574.8

(4-(2-(2-amino-4-oxo-4,7-dihydro-1H-pyrrolo[2,3-d]pyrimidin-5-yl)et

hyl)benzoyl)tyrosine(7)

To solid material (0.2 g, 0.348 mmol, 1 equiv), a solution of TFA/TIPS/H2O (95:2.5:2.5, 20 mL) was added. Upon completion product formation, the reaction mixture was cannulated as a steady stream to stirred methyltert-butyl ether (MTBE,200mL) at 23°C to give blue precipitate of compound. The precipitate was filtered using sintered funnel under aspirator vacuum, washed with MTBE (3×100 mL), and dried under high vacuum for 8 h to obtain compound(0.153 mg, 95.29%) as a blue solid. 1H-NMR (500 MHz, DMSO-d6/D2O)δ8.58 (s, 1H, Pyr−CH),7.76 (d,J= 8.2 Hz, 2H, Pte−Ar−CH), 7.56 (d,J= 8.2 Hz, 2H, Pte−Ar−CH), 7.02 (d,J= 8.3 Hz, 2H, Tyr−Ar−CH), 6.62−6.55 (m, 2H, Tyr−Ar−CH), 5.13−5.02 (m, 2H, Pte-CH2), 4.35 (dd,J= 9.5, 4.3 Hz, 1H, Tyr-αCH), 3.07 (dd,J= 13.8, 4.3 Hz, 1H, Tyr-CHH), 2.87 (dd,J= 13.8, 9.4 Hz, 1H, Tyr−CHH).13C-NMR (125 MHz, DMSO-d6/D2O)δ174.48, 164.86, 161.30, 156.60, 155.84, 155.44, 154.70,149.26, 143.87, 141.33, 135.28, 130.08, 129.15, 128.78, 128.19, 128.13, 117.33, 115.03, 114.81, 55.81, 53.90, 36.27. HRMS (ESI) calcd for C25H20F3N7O6[M+ H]+m/z461.1506; found:m/z 461.17.

4-(2-((E)-2-((E)-2-(4-(2-(4-(2-(2-amino-4-oxo-4,7-dihydro-1H-pyrrolo[2,3-d]pyrimidin-5-yl)ethyl)benzamido)-2-carboxyethyl)phenoxy)-3-(2-((E)-3,3-dimethyl-5-sulfo-1-(4-sulfobutyl)indolin-2-ylidene)ethylidene)cyclohex-1-en-1-yl)vinyl)-3,3-dimethyl-5-sulfo-3H-indol-1-ium-1-yl) butane-1-sulfonate(8)

Compound 4 (100 mg, 0.216 mmol, 1 equiv) was dissolved in water (5 mL) at 23°C, and pH of the solution was increased to ca. 9.5 by adding aqueous 3.75 M NaOH dropwise to give a clear blue solution. and pH of the solution was maintained at 9.5.To a solution of S0456 (182 mg, 0.205 mmol, 0.950 equiv) in water (2 mL) at 23°C, a solution of Compound 4 at pH 9.5 was added dropwise. The temperature of the reaction mixture was increased to 90°C, stirred at 90°C for 45 min. Upon completion of product formation, the reaction mixture was cooled to room tempeature and lyophilized. The crude material was further purified using prep-HPLC. The purity of compound 5 at wavelength 270 nm was ⩾97%. Analytical UPLC: Rt= 2.33 min [solvent gradient: 0% B to 50% B in 5 min]. UV: 225, 275, 350 nm.1H NMR (500 MHz, DMSO-d6/D2O)δ8.27 (s, 1H), 7.72 (d,J= 13.9 Hz, 2H), 7.66−7.56 (m, 4H), 7.41 (d,J= 8.3 Hz, 2H), 7.29 (d,J= 8.3 Hz, 2H), 7.19 (d,J= 8.2 Hz, 2H), 6.95 (d,J= 8.2 Hz, 2H), 6.53 (d,J = 8.3 Hz, 2H), 6.15 (d,J= 14.1 Hz, 2H), 4.42−4.29 (m, 3H), 4.08 (t, J= 7.2 Hz, 4H), 3.17−3.03 (m, 2H), 2.96 (dd, J= 13.8, 8.9 Hz, 1H), 2.75−2.58 (m, 5H), 2.55 (dd, J= 13.4, 6.5 Hz, 5H), 1.88 (s, 4H), 1.80−1.65 (m, 8H), 1.10 (d, J= 14.3 Hz, 12H).13C NMR (125 MHz,DMSO-d6/D2O)δ174.00, 171.64, 165.44, 163.11, 161.57, 157.90, 153.85, 150.54, 149.08, 148.41, 144.54, 142.31, 140.97, 140.40, 132.68, 131.02, 128.49, 127.73, 126.28, 122.39, 119.64, 113.98, 111.22, 110.54, 100.66, 54.43, 50.75, 48.49, 45.84, 43.75, 35.86, 27.25, 27.09, 25.95, 23.79, 22.49, 21.30, 20.83. HRMS (ESI) calcd for C61H67N9O17S4[M + H]$^+$m/z1312.51; found: m/z1313.10, [M +2 H]2$^+$ m/2z655.61.

^{1}HNMR Spectra

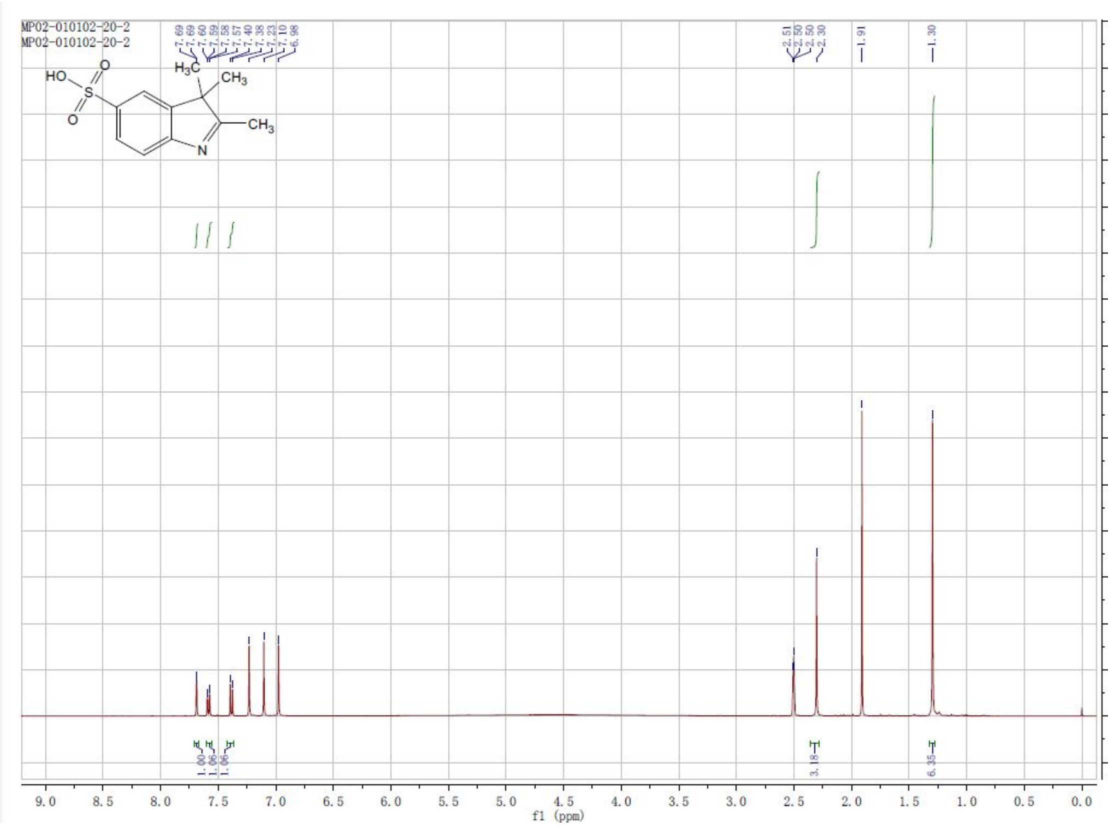

Fig. S1. ^{1}HNMR spectrum of 2 in DMSO-*d*6.

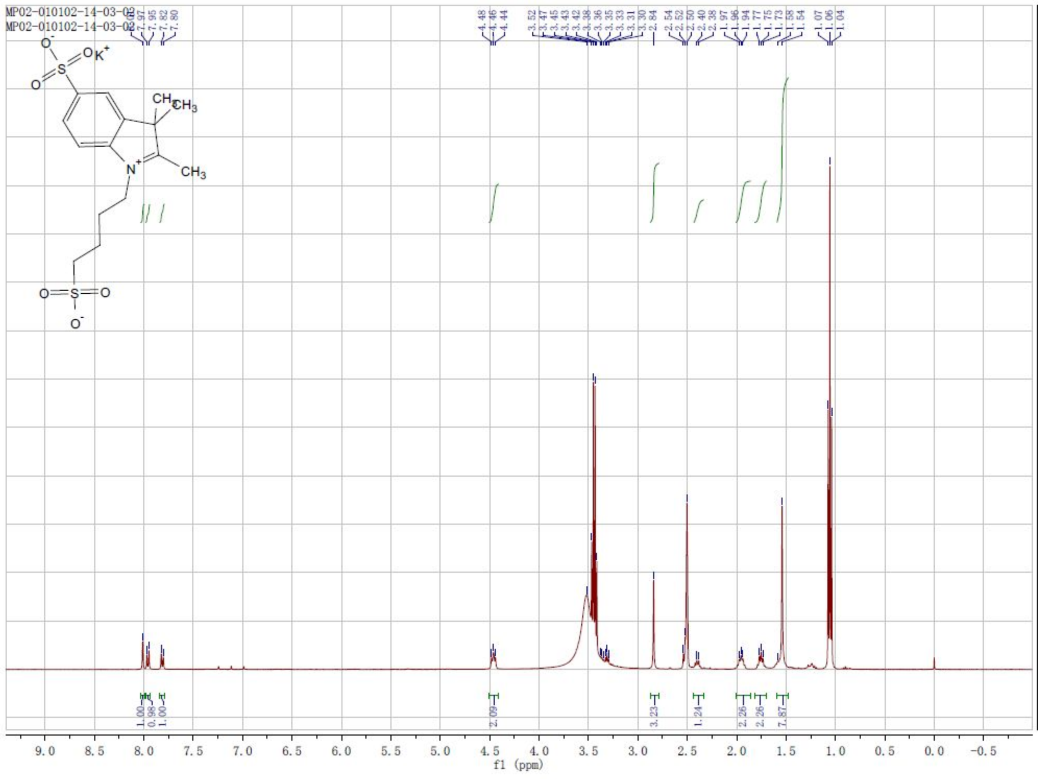

Fig. S2. ^{1}HNMR spectrum of 3 in DMSO-*d*6.

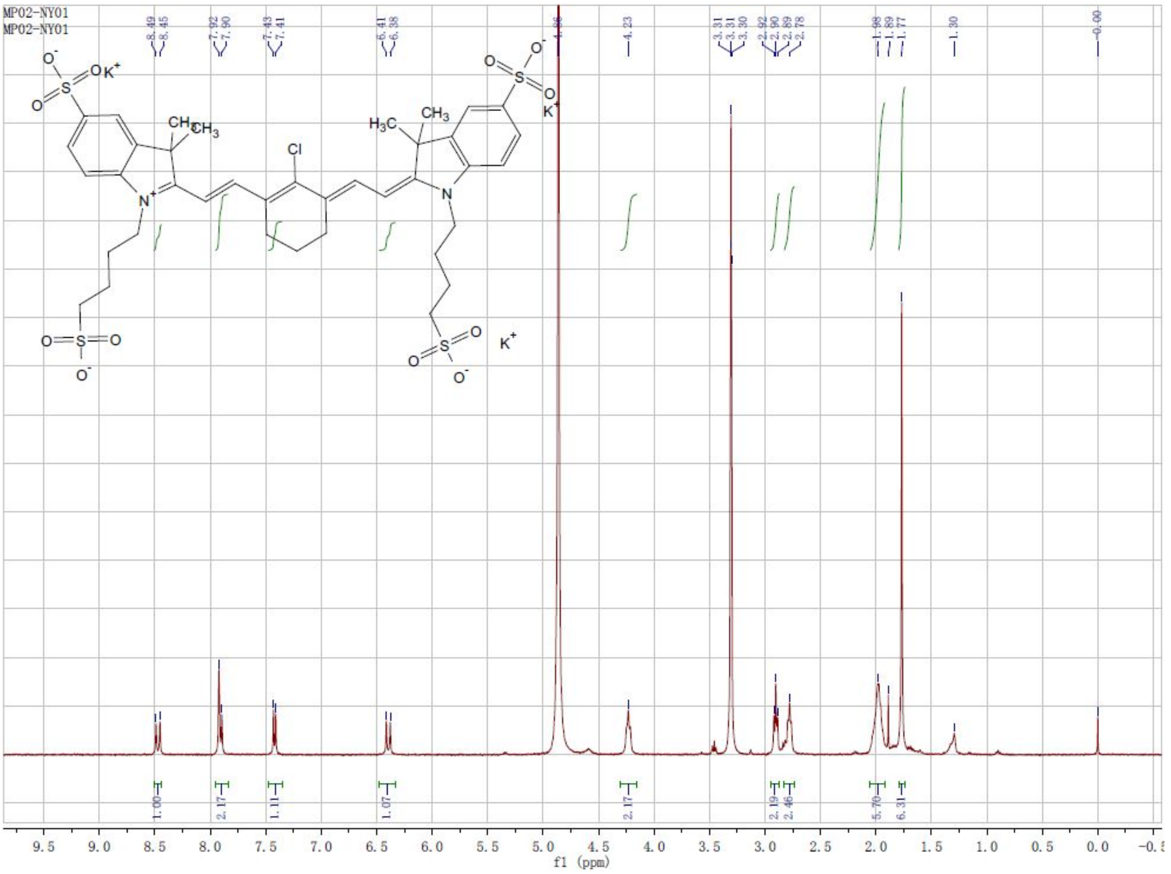

Fig. S3. ^{1}HNMR spectrum of 4 in DMSO-*d*6.

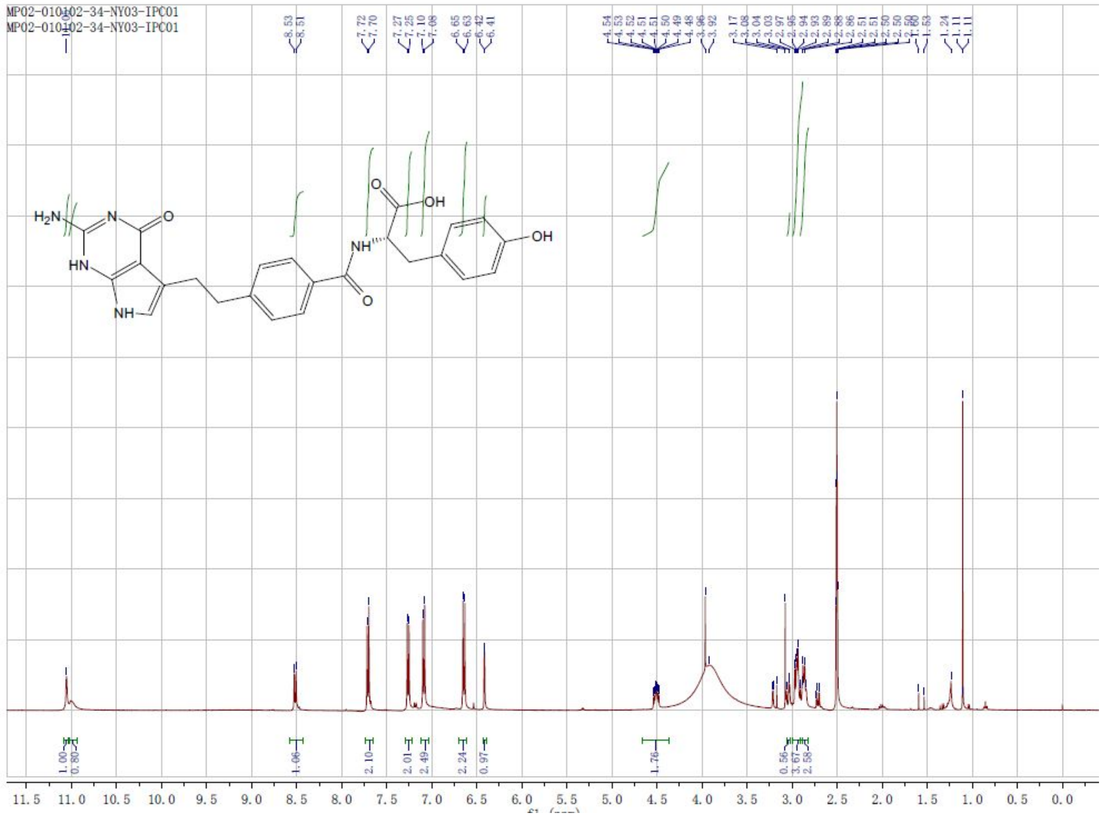

Fig. S4. ^{1}HNMR spectrum of 7 in DMSO-*d*6.

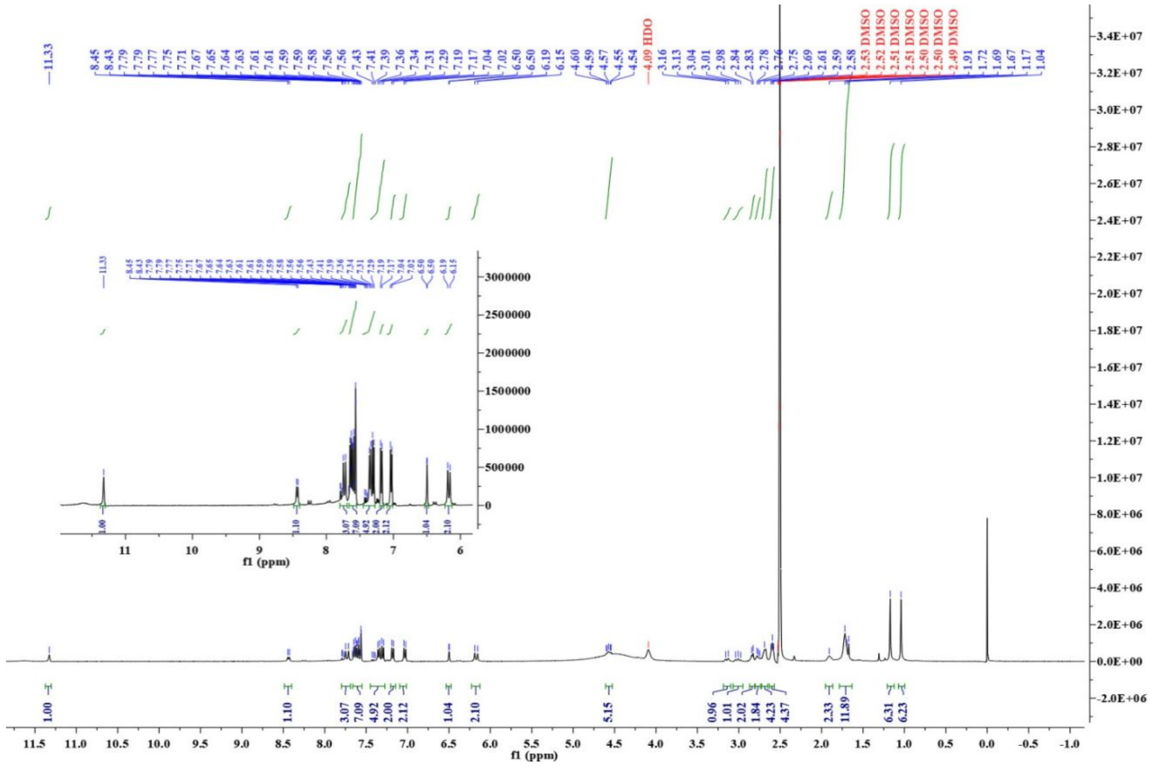

Fig. S5. ^{1}HNMR spectrum of NY-07 in DMSO-*d*6.

HPLC Spectra of NY-07

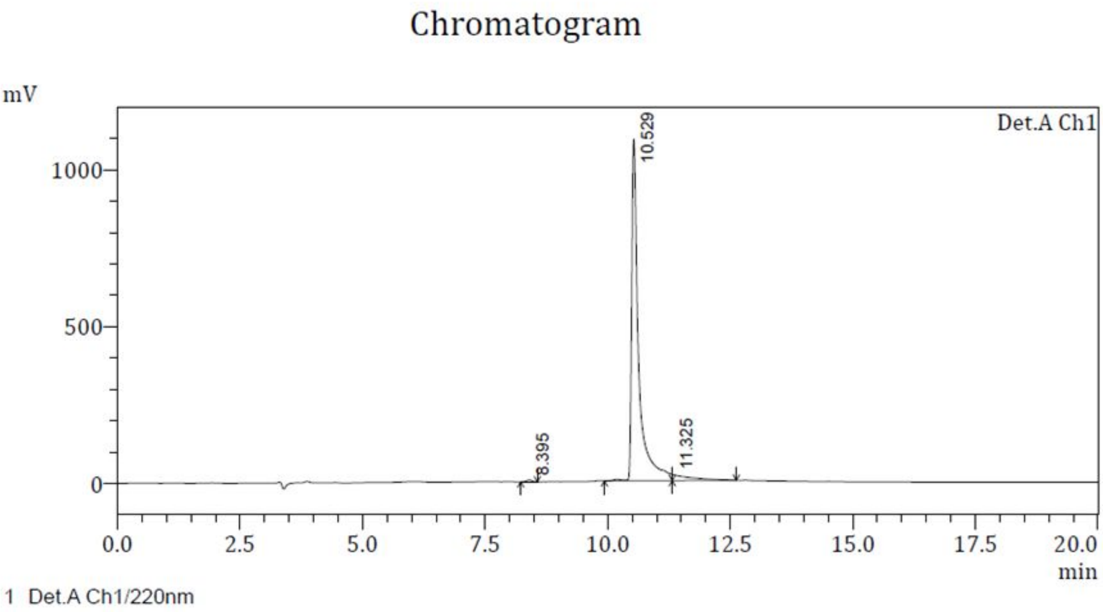

PeakTable

Detector A Ch1 220nm

Peak#	Ret. Time	Area	Height	Area %	Height %
1	8.395	44922	5896	0.397	0.529
2	10.529	10762338	1089961	95.165	97.759
3	11.325	501884	19089	4.438	1.712
Total		11309144	1114947	100.000	100.000

Fig. S6. HPLC analysis of NY-07.

MS analysis of NY-07

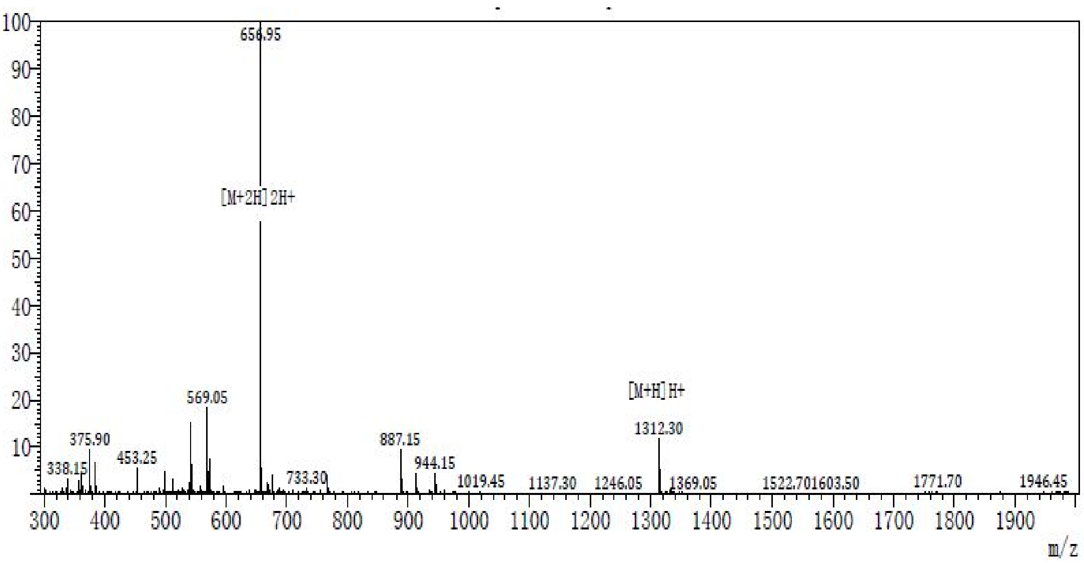

Fig. S7. MS analysis of NY-07.

Summary of Fluorescence performance

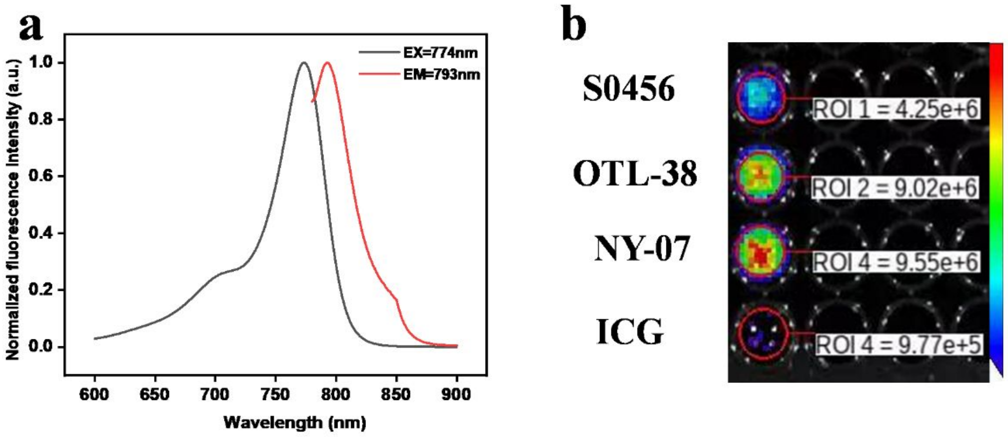

Fig. S8. Summary of Fluorescence performance. (a) Absorption spectra and emission spectra of 1nM NY-07 in 10 mmol/L PBS (pH = 7.4). (b) Fluorescence intensity of 1nM NY-07, S0456, ICG, OTL-38.

Compare the stability of NY-07 and OTL-38 in PBS solution

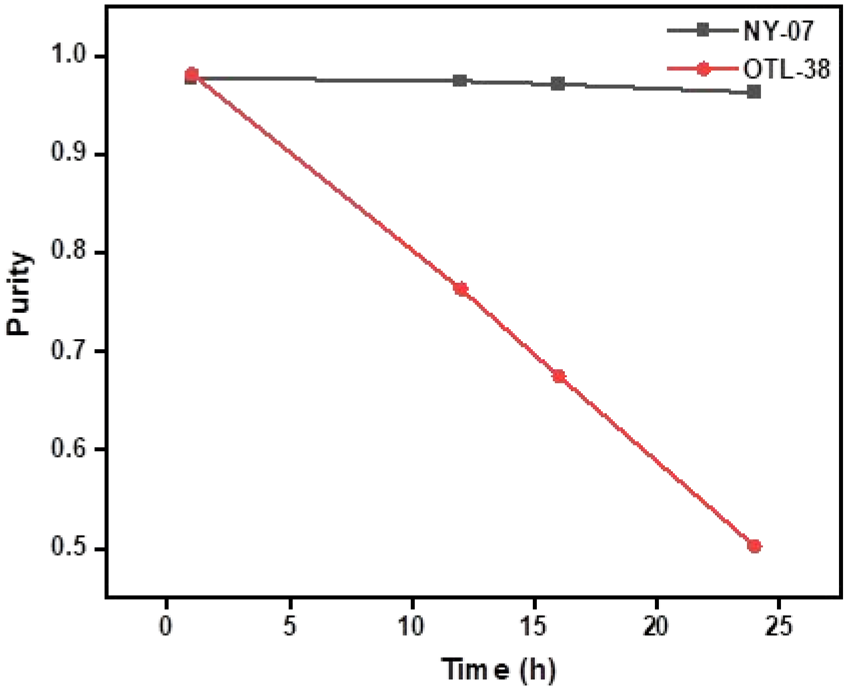

Fig. S9. Compare the stability of NY-07 and OTL-38 in PBS solution.

Docking studies of NY-07 OTL-38 Pemetrexed and Folic

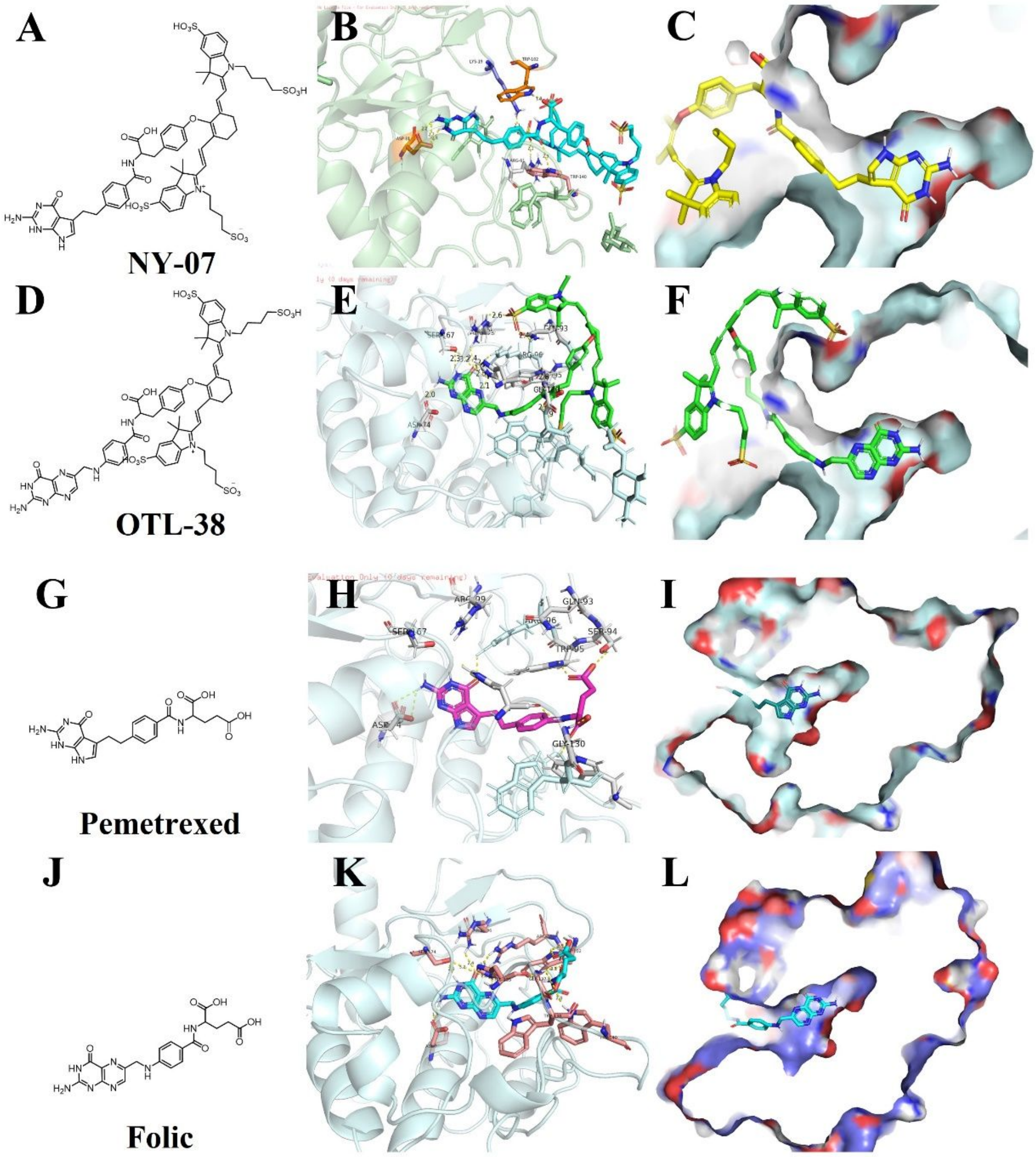

Fig. S10. Docking studies of NY-07 OTL-38 Pemetrexed and Folic. A, Chemical structures of NY-07. B, Molecular dynamics prediction of NY-07 (blue) and the binding interaction of the molecule at the active site of FRα (PDB code: 4LRH). C, Side view of FRα and NY-07 in stick mode. Proteins are depicted in a ribbon cartoon pattern and side chains are depicted in a silkscreen pattern with standard three-letter amino acid codes. D, Chemical structures of OTL-38. E, Molecular dynamics prediction of OTL-38 (blue) and the binding interaction of the molecule at the active site of FRα (PDB code: 4LRH). F, Side view of FRα with OTL-38 in stick mode. Binding pocket of FRα in space-filling mode. Green and yellow are carbon atoms,

blue are nitrogen atoms, and red are oxygen atoms. G, Chemical structures of Pemetrexed. H, Molecular dynamics prediction of Pemetrexed (blue) and the binding interaction of the molecule at the active site of FRα (PDB code: 4LRH). I, Side view of FRα and Pemetrexed in stick mode. Proteins are depicted in a ribbon cartoon pattern and side chains are depicted in a silkscreen pattern with standard three-letter amino acid codes. J, Chemical structures of Folic K, Molecular dynamics prediction of Folic (blue) and the binding interaction of the molecule at the active site of FRα (PDB code: 4LRH). L, Side view of FRα with Folic in stick mode. Binding pocket of FRα in space-filling mode. Green and yellow are carbon atoms, blue are nitrogen atoms, and red are oxygen atoms.

In vivo efficacy and specificity of NY-07

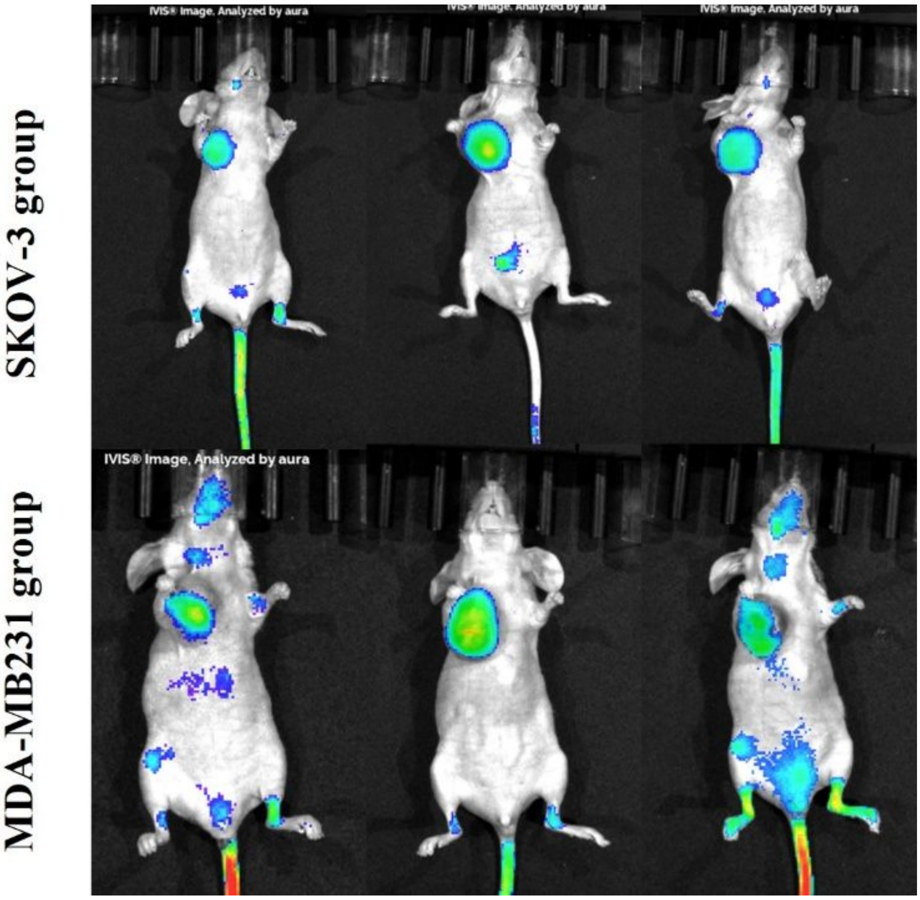

Fig. S11. In vivo efficacy and specificity of NY-07. Representative IVIS images showing overlay of fluorescence images over white light images of mice bearing FRα + SKOV-3 tumors, MDA-MB-231 tumors.

Quantitative analysis of fluorescence intensity of isolated organs

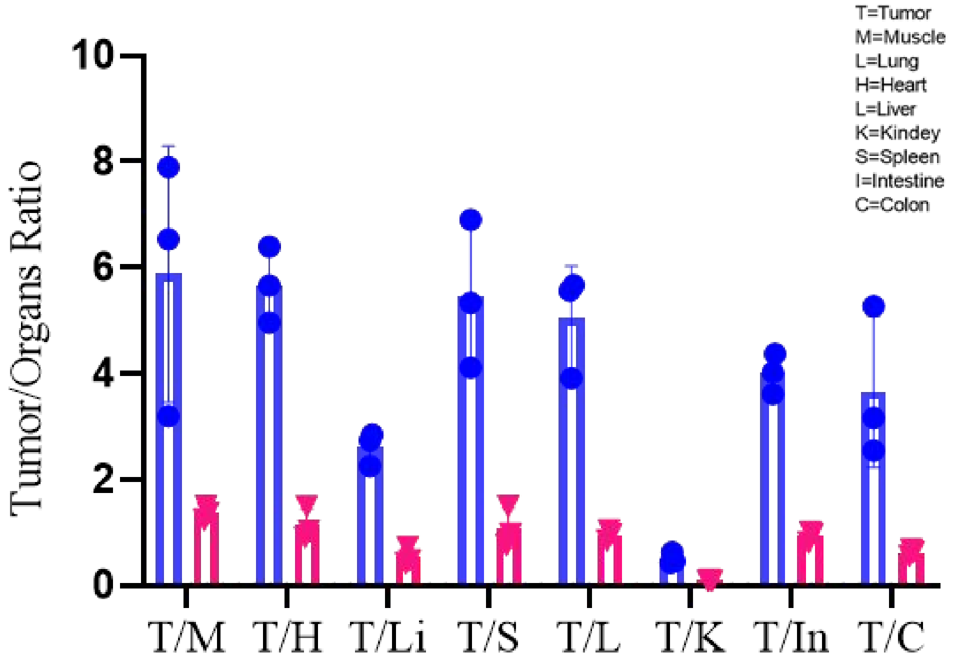

Fig S12. Quantitative analysis of fluorescence intensity of isolated organs.

NY-07 accumulates in FRα^{+} NCI-H1299 Xenografts

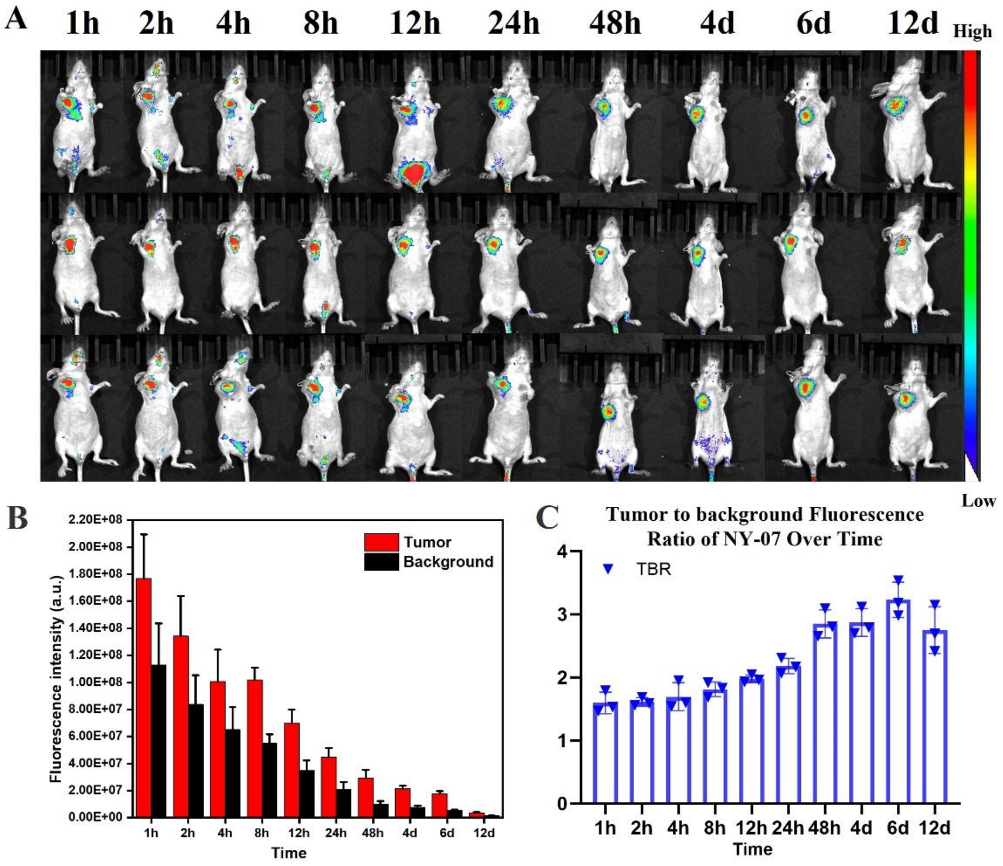

Fig S13. NY-07 accumulates in FRα+ NCI-H1299 Xenografts. NCI-H1299 tumor bearing mices were administered NY-07 at 10nmol/per then imaged with the IVIS Imaging System. A, Representative images of mice at various times after intravenous drug delivery, B, Trends in mean fluorescence intensity of tumors and background in NCI-H1299 Xenografts models, C, Tumor to background ratio (TBR) was obtained and plotted over time from drug delivery.

Toxicity of NY-07 & folic acid (FA) toward L02 cell line

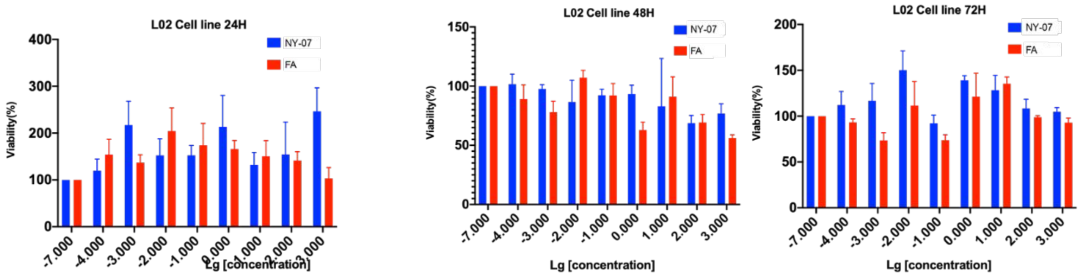

Fig S14. Toxicity of NY-07 & folic acid (FA) toward L02 cell line.

Long-term imaging of NY-07, OTL-38, ICG

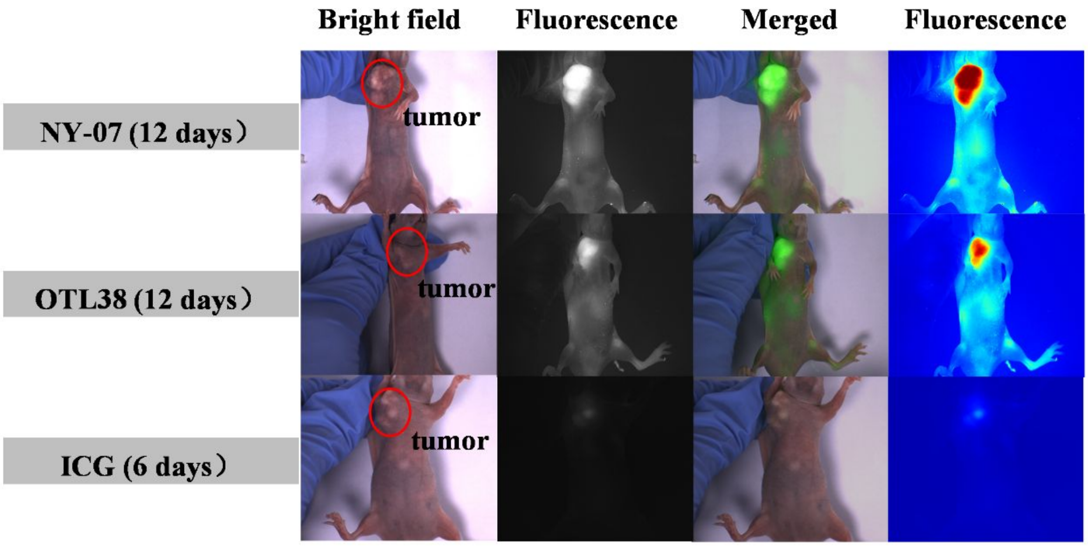

Fig S15. Long-term imaging of NY-07, OTL-38, ICG.

Localization of NY-07 in liver microtumors

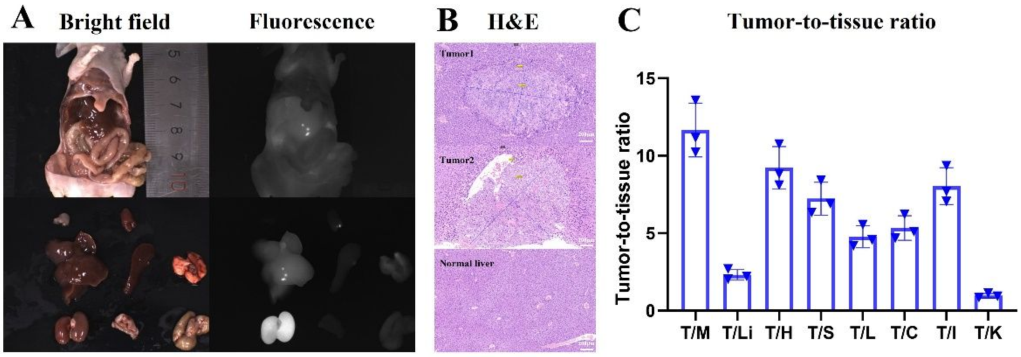

Fig S16. Localization of NY-07 in liver microtumors. A, The Balb/c nude in vivo and ex vivo imaging at 24 h post-injection NY-07, left: white light, right: fluorescence. B, H&E staining of liver cancer. C, Analysis of fluorescence intensity ratio of microtumors and organs. T: tumor M: muscle Li: liver H: heart S: spleen L: lung C: colon I: intestine K: kidney. Tumor 1: Long diameter 653μm and short diameter 1156.9μm. Tumor 2： Long diameter 765.1μm and short diameter 552.1μm. Scale bar=200μm.

In vivo efficacy and specificity of probe NY-07 in colorectal cancer mice model

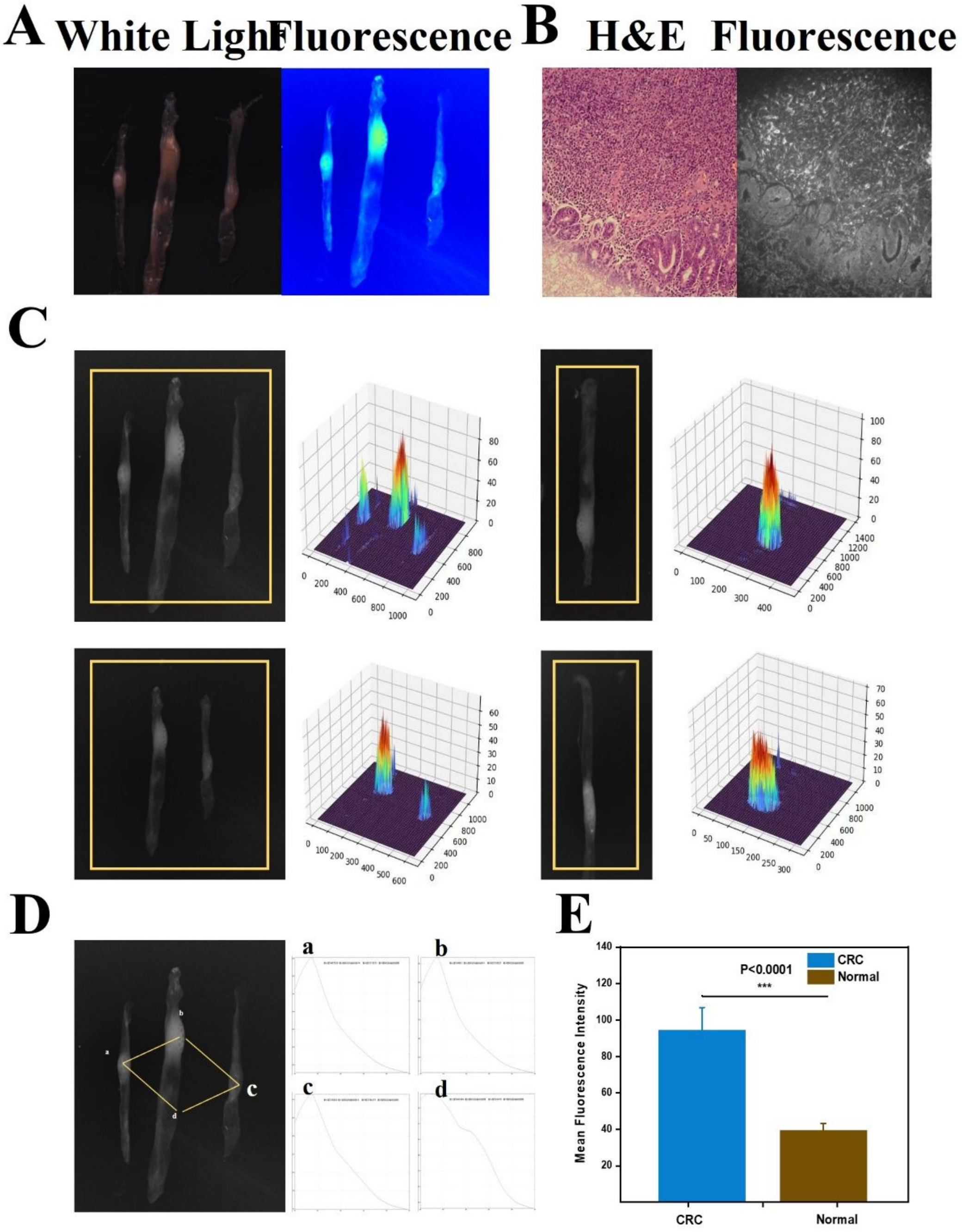

Fig S17. In vivo efficacy and specificity of probe NY-07 in colorectal cancer mice model. A, The excised colon imaging at 12 h post-injection, left: white light, right: fluorescence. B, H&E staining of colorectal cancer and fluorescence image. C, Area analysis and the interactive 3D

surface plot of the gray value within box. D, Use the spectral pen to detect the fluorescence intensity at different locations. E, Quantification of mean fluorescence intensities of the excised colons. Scale bar=200μm.
